# Evaluating the utility of large language models for detecting and simulating language dysfunction

**DOI:** 10.3389/frai.2026.1857106

**Published:** 2026-06-24

**Authors:** Yan Cong, Jiyeon Lee, Nalin Rajput, Emily A. Tumacder, Arianna N. LaCroix

**Affiliations:** 1 School of Languages and Cultures, Purdue University, West Lafayette, IN, United States; 2 Center on Aging and the Life Course, Purdue University, West Lafayette, IN, United States; 3 Department of Speech, Language, and Hearing Sciences, Purdue University, West Lafayette, IN, United States; 4 Department of Statistics, Purdue University, West Lafayette, IN, United States; 5 School of Electrical and Computer Engineering, Purdue University, West Lafayette, IN, United States

**Keywords:** aphasia, automatic clinical assessment, data quality, large language models, prompt engineering, translational medicine

## Abstract

This study investigates the utility of large language models (LLMs) in simulating and classifying language dysfunction, with a focus on aphasia. We conducted three studies to evaluate the extent to which LLMs can approximate surface-level linguistic features of individuals with aphasia and whether synthetic data can support classification tasks when real data is limited. In Study 1, we prompted an LLM to generate synthetic utterances and used human rating surveys to evaluate whether the resulting outputs capture surface-level features associated with agrammatic language production. Results showed that approximately half of raters were unable to reliably distinguish AI-generated from human-produced agrammatic speech. This pattern suggests that the synthetic data captured some surface-level features associated with agrammatic utterances; however, low inter-rater agreement indicates substantial rater uncertainty and task ambiguity—likely reflecting the difficulty of making reliable judgments from brief, text-only excerpts—limiting conclusions about clinical fidelity. In Study 2, we assessed the performance of (fine-tuned) LLMs on two binary classification tasks: (1) detecting agrammatic features and (2) classifying utterances based on an LLM-derived surprisal index (negative log-likelihood of a token given its context). We examined models’ successes and failures by comparing performance across training conditions and against a classical machine learning baseline (logistic regression) model. We found preliminary and suggestive evidence that the classical machine learning model remained superior for binary agrammatic utterances detection, however, fine-tuned LLMs showed advantages in approximating the LLM-derived surprisal index compared to their pre-trained counterparts and the baseline logistic regression model. We additionally conducted an exploratory analysis in Study 3, where we prompted an LLM for end-to-end aphasia severity prediction. Our overall results suggest that, as a proof-of-concept, transformer-based models, particularly when fine-tuned on curated synthetic data, can learn task-relevant patterns of language dysfunction. Our findings demonstrate the potential utility of LLMs in modeling not only language function (language produced by the general population) but also patterns of language dysfunction (such as those observed in aphasia), offering insights into the linguistic features of disordered language. We cautiously conclude that, with further development, LLMs may serve as useful tools for data evaluation and generation in the study of language dysfunction.

## 1 Introduction

Large Language Models (LLMs) offer new opportunities for psychological science by enabling scalable analysis and generation of human-like language data. These capabilities are particularly valuable for studying clinical populations, where limited data hinder understanding of disordered language systems. Aphasia, characterized by a broad range of speech and language deficits such as impaired fluency, word retrieval, and grammatical constructions, represents an ideal clinical population for this investigation due to its well-characterized language production patterns. Despite advances in computational linguistics, natural language processing (NLP), and machine learning (Salem et al., 2023; Liu et al., 2023; Corcoran et al., 2020; Rezaii et al., 2024; Tang et al., 2023; Cong et al., 2024a; Cong et al., 2024b; Cong and Lee, 2025, among others), tailoring pre-trained LLMs to aphasia research remains underexplored.

One major challenge in pre-training LLMs to identify language disorders such as aphasia is data scarcity. To address this challenge, we generated synthetic aphasia data using prompt engineering in GPT-4o-mini (OpenAI, 2024). The specific language impairment we targeted was agrammatism-related features, characterized by difficulty producing grammatical structures, often resulting in simplified speech with omitted function words (e.g., “is,” “the,” “at”). We then assessed the quality of this AI-generated synthetic data using human rating surveys. The goal of this evaluation was not to establish clinical fidelity, but rather to assess whether AI-generated utterances capture surface-level features associated with agrammatic language. We hypothesized that LLMs can generate synthetic data that captures surface-level features associated with agrammatic language.

The second aim of this study was to explore whether fine-tuned LLMs could identify agrammatic and language atypicality features associated with aphasic speech. We finetuned two LLMs, Llama (Grattafiori et al., 2024) and Gemini Flash 2.0 (Google, 2025: *gemini-2.0-flash-001*, February 05 version), using the AI-generated synthetic utterances produced by GPT-4o-mini in Study 1. We then asked Llama and Gemini to complete two tasks: (1) detect the presence of agrammatic utterances, and (2) classify utterances based on an LLM-derived surprisal index (negative log-likelihood of a token or sequence of tokens given its context). These tasks were conducted on both real and synthetic datasets to assess and compare model performance. We hypothesized that LLMs fine-tuned with data augmentation would more effectively detect surface-level agrammatic features and more accurately classify the surprisal-based index of linguistic atypicality than models without such synthetic data augmentation. In this context, synthetic data does not introduce new clinical information, but rather amplifies patterns of language atypicality present in the training distribution via a larger, augmented dataset, exposing models to more data and learning cases.

Besides systematically examining the role of data and training mechanisms on models’ performance in detecting and simulating aphasia, in Study 3, we additionally conducted an exploratory analysis on prompting a pre-trained LLM to detect discrete categories of aphasia severity, moving beyond binary detection and towards more clinically relevant detection.

We emphasize that the present study should be interpreted as a proof-of-concept investigation rather than a definitive clinical validation. We focused on computational feasibility, with the hope that our proposed methods and pipelines could inform clinical applicability in the future. Our contribution is primarily methodological (providing pipelines and approaches to evaluating the utility of LLMs for detecting and simulating language dysfunction), rather than conceptual or clinical. The hope is that future clinical questions could be informed using the proposed methods and pipelines. To promote reproducibility, we have made our code and metadata publicly available in this OSF repository: 10.17605/OSF.IO/DRMCY.

In general, our work concerns AI in translational medicine, particularly its emphasis on data quality, validation challenges, and the responsible implementation of AI in clinical science. The study addresses a core translational challenge of the scarcity of high-quality clinical data by exploring whether LLMs can generate synthetic speech data that approximates the linguistic features of aphasia, a neurological language disorder, and whether fine-tuned LLMs can reliably classify such disordered language. In doing so, it directly engages themes the call highlights: what AI can generate in translational pipelines, the critical importance of rigorous validation, and the risks of bias and overgeneralization in AI models applied to patient populations. Our methodological contributions, including synthetic data generation, fine-tuning pipelines, and human validation frameworks, offer generalizable approaches relevant to general challenges regarding data scarcity and validation in AI-driven translational research.

## 2 Background

### 2.1 Previous work on synthetic data and simulation

Recent studies have discussed the potential of synthetic data in addressing challenges in aphasia research and clinical applications (Bedrick et al., 2025; Hux et al., 2017). Salem et al. (2023) recently used the BigBird model to automate the prediction of the target word when a paraphasia, or word substitution error, was made. Their model’s performance was validated by determining the alignment between its predictions and human identifications, with higher alignment indicating better performance. Using 332 transcripts from people with aphasia (PWA) and 256 from controls, the fine-tuned BigBird model achieved an accuracy of 50.7% in precisely matching the human-identified target, and 70% in the top five predictions scenario, where the human-identified target word was among the top five words predicted by the model. Despite these promising results, synthetic data from controls did not improve model performance, likely due to quality issues, among other factors. Additionally, BigBird showed challenges in handling nonword paraphasias and phonetic transcriptions. Gale et al. (2023) extended this work when they introduced BORT, a model requiring both orthographic and phonetic representations to address the limitations of text-dominant models like BigBird. By using the International Phonetic Alphabet (IPA) alongside orthography, BORT showed promise for analyzing aphasic speech errors, with high accuracy and low CER rates (i.e., Character Error Rate, which is calculated by dividing the number of *character* errors in a target word by the length of that word, where a character can be a single letter, number, symbol, or space within a text string), indicating multiple input parameters representing the same dataset increases model performance.

Relatedly, Misra et al. (2023) attempted to address data scarcity by generating synthetic aphasic utterances, combining neurolinguistics and NLP to create large datasets based on linguistic features such as mean length of utterance and noun-to-verb ratio. The T5 language model fine-tuned on these datasets was assessed using classic measures about the quality of machine-generated text, such as the BiLingual Evaluation Understudy score (BLEU, Papineni et al., 2001). The model achieved a high score of 0.827 in BLEU. The model’s performance on the testing dataset was also evaluated using similarity of predicted and intended sentences, with a high score of 0.904, demonstrating the potential utility of using synthetic data in aphasia research and the development of communication-assistive tools.

Li et al. (2023) proposed two clinical data generation methods using LLMs: *data-to-label,* which annotates existing datasets, and *label-to-data*, which generates synthetic sentences based on predefined labels. These methods emphasize the hierarchical quality of datasets, which can enhance LLM performance in clinical prediction tasks. Belgoumri et al. (2024) used ChatGPT to generate synthetic parallel corpora for French sentences affected by Broca’s aphasia, achieving a BLEU score of 0.796. Vechkaeva and Neumann (2020) applied a data generation approach called generative adversarial networks (GANs) for aphasia classification, showing that GANs could improve classification accuracy, though noise in utterance-level datasets posed challenges. These studies suggest that synthetic data can enhance models’ performance in aphasia-related tasks.

Notably, while these efforts to address data scarcity have reported high automated performance metrics, such as the BLEU scores of 0.827 in Misra et al. (2023) and 0.796 in Belgoumri et al. (2024), these scores likely reflect *n*-gram overlap rather than true clinical fidelity. As noted by Bedrick et al. (2025), adequate evaluation of synthetic clinical data requires a more rigorous typology that distinguishes between mere linguistic resemblance and the replication of diagnostic markers. It is possible that a high BLEU score indicates fluent text generation, but it is also likely that it masks a potential failure to simulate the specific linguistic atypicality required for clinical research. Following this line of work (e.g., Bedrick et al., 2025; Salem et al., 2023), we proposed to design validation studies that involve assessing the alignment between model outputs and human expert judgment, as (fully) automated metrics can fail to capture the nuanced, context-dependent manifestations of language dysfunction.

Against this background, we proposed that task-specific fine-tuning can offer a practical approach to leveraging LLMs in aphasia research, as it required considerably less data than the original (pre-)training phase, making it more feasible in contexts where aphasia (or other language dysfunction) data is limited. We highlighted the novel intersection of synthetic data generation, fine-tuning on PWA data, and task-specific evaluations. This intersection has yet to be thoroughly explored in aphasia research. We maintain that this integration of approaches can be promising for advancing research at the intersection of computational linguistics and psychological research, as well as the eventual automation of aphasic discourse data analysis, which may ultimately improve diagnostic and therapeutic strategies for language dysfunction.

### 2.2 LLM-surprisals and aphasia research

LLM-surprisal scores have been used to quantify and understand the unpredictability of aphasic language at both the word and sentence levels (Rezaii et al., 2023; Cong et al., 2024a, 2024b). Prior research by Cong et al. (2024a) demonstrated that high surprisal scores identified utterances that deviated from the “typical” language patterns predicted by the model, reflecting unexpected word choices or sentence structures. They further showed that individuals with Broca’s aphasia showed significantly higher surprisal scores compared to those with Wernicke’s aphasia, suggesting that LLM-surprisals effectively captured linguistic patterns aligned with clinical markers of agrammatic utterances, through a probabilistic, data-driven framework that could potentially enrich clinicians’ observational judgment.

Inspired by this line of investigation, we maintain that the surprisal index could offer a scalable and objective means to detect subtle language dysfunction in aphasia. Concretely, our current work utilizes LLM-surprisals as one of the labels in our pipeline. Labels are crucial for operationalizing the detection and simulation of language dysfunction in aphasia. To facilitate labeling, we used the clinical annotations for agrammatic utterances from the AphasiaBank (https://talkbank.org/aphasia/; https://talkbank.org/aphasia/examples/index.html; Macwhinney and Fromm, 2023; MacWhinney et al., 2011). We additionally introduced a synthetic labeling approach by transforming LLM-derived surprisal scores into categorical labels, as proxies for identifying aphasic and atypical language utterances.

Clinical annotations of agrammatic utterances rely on expert judgment of observable speech characteristics, such as reduced utterance length and complexity and the omission of function words. In contrast, surprisal-based labels can potentially provide a quantitative, holistic measure that integrates lexical choices and structural deviation (Cong et al., 2024a, 2024b). For example, surprisal scores can capture more nuanced disruptions, such as the use of high-frequency content words and simplified syntax, common to agrammatic utterances (Rezaii et al., 2023). Incorporating surprisal-based measures provides complementary insights into both word-level and structural anomalies, potentially offering a more integrated view of aphasic speech.

## 3 Method

### 3.1 Study 1: generate and evaluate synthetic data

#### 3.1.1 Source of the main pipeline: existing, real data

For the main analyses in Study 1 and 2, we obtained transcripts from 279 PWA (110 females, age: mean 61.75, range 25.07–90.08; SD 12.49) and 341 Healthy Controls (HC, 229 females; age: mean 50.92; range 18.00–89.00; SD 21.38) from the AphasiaBank who were English speakers (Macwhinney and Fromm, 2023; MacWhinney et al., 2011). Aphasia type and severity were characterized using the Western Aphasia Battery-Revised (WAB-R) (Kertesz, 2006). The participants’ aphasia severity, measured by the WAB-R Aphasia Quotient (AQ), had a mean of 71.52 (SD = 19.97) with a range from 10.8 to 99.6, and their spontaneous speech fluency averaged 6.33 (SD = 2.5) on a scale of 0 to 10. Regarding aphasia type, 31.7% were classified as Anomic, 21.2% as Broca’s, 8.6% as Wernicke’s, 16.9% as Conduction, 9.7% were not aphasic using the WAB-R criteria, and 11.9% fell into other categories including Global, Transcortical Motor, and Transcortical Sensory aphasia.

We extracted transcripts from two tasks: (1) Cinderella story narrative retell, which involved participants reviewing a wordless picture book before retelling the story of Cinderella without looking at the book; and (2) Picture descriptions, where participants described pictures about a broken window, refused umbrella, and cat rescue.

For the exploratory analysis in Study 3, we curated a different dataset from the AphasiaBank, with details provided in Section 3.3.

#### 3.1.2 Two labels: agrammatic and LLM-surprisals indexing linguistic atypicality

We constructed synthetic data using two labeling frameworks. Specifically, we generated AI (i.e., LLM)-produced, synthetic utterances conditioned on predefined labels corresponding to (1) an agrammatism-related label of agrammatic utterances and (2) a surprisal-derived index of linguistic atypicality. Here, the labels define the target characteristics to be represented, while the synthetic data consist of the AI-generated utterances produced to reflect those characteristics. Notably, all LLMs in this paper were accessed via API (Application Programming Interface) for the open-source Llama^1^ and closed-source Gemini API^2^ rather than a chatbot interface, in order to improve robustness, reproducibility, and patient privacy protection.

First, from the AphasiaBank transcripts, we used the existing AphasiaBank labels for agrammatism,^3^ which are annotated by clinicians in CHAT format “[+ gram].” We focused on agrammatism for both clinical and practical considerations. Clinically, agrammatism is a key linguistic feature that distinguishes non-fluent from fluent aphasia. Practically, the CHAT framework provides computationally consistent annotations of agrammatic utterances. This enabled the efficient identification and extraction of agrammatism from real data, which also facilitated the generation and validation of the synthetic data.

The second labeling approach involved creating a surprisal-based categorical label computed using GPT-2 (Radford et al., 2019). Building upon the findings of Cong et al. (2024b) as well as Cong et al. (2024a), which demonstrated that LLM-surprisals can serve as a reliable metric for detecting core agrammatic features at both the word and sentence levels, we proposed an extension of this methodology. We hypothesized that LLM-surprisals could serve as a proxy for a composite, utterance-level measure of linguistic atypicality, capturing distributed deviations from expected, “typical” language patterns, rather than directly constituting a clinical label of aphasic speech per se. We applied this label derived from LLM-surprisals to synthetic as well as real datasets and incorporated fine-tuning methodologies, marking a departure from Cong et al. (2024a), which relied solely on real datasets and pre-trained LLMs.

Surprisal values for each utterance were calculated following the procedures outlined by Cong et al. (2024a) and then normalized by utterance length to produce weighted surprisal scores, preventing longer texts from disproportionately influencing the overall mean. These weighted surprisal scores were transformed into categorical labels consistently. Specifically, utterances with weighted surprisal scores above the overall mean (as grouped by participant and task) were assigned a label of 1, indicating LLM-surprisal indexed atypical, aphasic-like language, while those below the mean were labeled 0, indicating typical, non-aphasic-like language. The distribution of raw GPT-2 surprisal scores can be found in the Supplementary material S5.

#### 3.1.3 Generating new, synthetic data

We used prompt engineering to generate synthetic agrammatic data (i.e., AI-generated utterances). We selected GPT-4o-mini (OpenAI, 2024), because of autoregressive LLMs’ decent performance in text generation (Tunstall et al., 2022; Jurafsky and Martin, 2025). We crafted prompts (Supplementary materials S1) based on the AphasiaBank (MacWhinney et al., 2011; MacWhinney and Fromm, 2023). The prompt begins with a task description and definition of agrammatic utterances that comes from the AphasiaBank, which offers a concise and sufficiently informative definition for the LLM to learn (source: https://talkbank.org/aphasia/examples/index.html: *“Speech that is reduced in length and/or complexity; function words and morphemes may be missing”*. Namely, for example, omission of function words, simplified morphology). Detailed instructions were provided, including example utterances annotated as “agrammatic,” identified using the CHAT notation “[+ gram]” from the AphasiaBank transcripts. We explored zero-shot prompting without examples (Supplementary material S1, Variant 1) and shorter prompts without specific output format instructions (Supplementary material S1, Variant 2), among other prompt variations (Supplementary material S1, Variants 3, 4). The Supplementary material S1, Final version prompt was the most effective, according to a qualitative, manual quality check conducted by an author who is a trained computational linguist. This manual check was a pilot-stage qualitative task used to filter out ineffective prompts; no numerical metrics were used. During this manual check, effectiveness was judged based on whether the model outputs consistently exhibited the targeted aphasia-like features, with proxy criterion described by the AphasiaBank (MacWhinney et al., 2011; MacWhinney and Fromm, 2023). Prompts leading the AI-generated language that was too fluent or generic were considered as less effective in capturing the specific pathological markers required, hence they were discarded. Concretely, we decided this by manually comparing AI-generated language with what the AphasiaBank described as agrammatic utterances “*Speech that is reduced in length and/or complexity; function words and morphemes may be missing*” (https://talkbank.org/aphasia/examples/index.html; MacWhinney et al., 2011; MacWhinney and Fromm, 2023), and prompts were rejected if the resulting AI-generated language did not mirror the pathological markers documented in the AphasiaBank.

We prompted GPT-4o-mini with the selected prompt (Supplementary material S1, Final version), asking it to generate synthetic agrammatic utterances and the corresponding non-agrammatic utterance. In total, GPT-4o-mini generated 6,000 pairs of synthetic agrammatic utterances and their non-agrammatic targets. Including both agrammatic and non-agrammatic synthetic data was important for the validation process, as it allowed us to explore the quality of both synthetic forms.

The generation of AI-generated data based on LLM-surprisal differed from that of synthetic agrammatic data, as the two labeling approaches capture different aspects of language dysfunction. As described, the agrammatic label was extracted from clinicians’ annotations from the AphasiaBank and targets grammatical impairments (MacWhinney et al., 2011; MacWhinney and Fromm, 2023, e.g., omission of function words, simplified morphology). On the other hand, the surprisal-based label was computed from utterance-level LLM-surprisal scores, reflecting deviations from expected language patterns that may extend beyond agrammatic features. Accordingly, we prompted GPT-4o-mini to generate synthetic utterances corresponding to low- and high-surprisal conditions, representing relatively typical, non-aphasic and atypical, aphasic-like language patterns, respectively.

Under this framework, high-surprisal utterances that are not labeled as agrammatic may exhibit more subtle irregularities—such as lexical retrieval difficulties, disrupted fluency, or unexpected word choices—that increase surprisal without necessarily violating grammatical structure. Thus, the surprisal-based data captures a broader range of linguistic atypicalities, whereas the agrammatic data isolates grammar-specific features. We therefore treated these datasets as complementary: the agrammatic set supports targeted learning of a well-defined feature, while the surprisal-based set enables the modeling of more diffuse patterns of language atypicality. This design allowed us to test whether incorporating both label types improves models’ ability to learn and discriminate between different forms of atypical language.

#### 3.1.4 Validating synthetic data

We designed two surveys to validate the quality of the synthetic data, inspired by Salem et al. (2023). First, as a sanity check and a preliminary prompt-adherence verification, two authors who are both certified speech-language pathologists (SLP) trained the other author and a student research assistant (RA) to identify agrammatism in aphasic speech. This author and the research assistant also accessed web-based materials from the AphasiaBank to further augment their understanding of agrammatism. Using an offline survey, the author and the RA each judged whether a synthetic utterance was agrammatic (1) or not (0), and provided a confidence rating from 1 (not confident) to 5 (very confident). Since all the synthetic agrammatic utterances were hypothesized to be agrammatic, a higher proportion of “yes, agrammatic” responses coupled with greater confidence would indicate better AI-generated synthetic data quality. We used this proportion as a proxy metric for validating the synthetic data. For this sanity check, we approximated that proportions exceeding 50% indicated better than chance quality, while proportions below 50% suggested lower than chance quality. Conversely, proportions closer to 100% indicated good quality. In total, half of the AI-generated synthetic dataset (3,000 out of 6,000 utterances) was evaluated in this offline survey. This sanity check was initial and not an independent assessment.

Our primary validation relied on a second, web-based survey involving raters unfamiliar with the project’s internal prompting strategies, providing a more robust measure of data quality than the initial internal check. Following Salem et al. (2023) and Jones and Bergen (2025), we designed this independent survey to examine whether certified SLPs and trained student RAs who were unfamiliar with the project could reliably detect agrammatism and distinguish AI-generated synthetic speech from speech produced by actual people with aphasia, effectively a Turing test paradigm. At the outset of the web-based survey, 12 participants answered background questions about clinical experience and proficiency in identifying agrammatism in individuals with aphasia. Participants reported the following years of clinical experience working with individuals with aphasia: one participant reported 25 years of experience, one reported 5 years, three reported 1 year, one reported 0.5 years, and six reported 0 years of experience; these six participants reporting 0 years were likely student RAs in aphasia research laboratories. Thus, they may have been familiar with agrammatic speech characteristics despite lacking formal clinical training. To this end, when asked whether participants considered themselves proficient in identifying agrammatism in individuals with aphasia, all participants, including those with 0 years of clinical experience, indicated either “Yes” or “Relatively proficient.” These 12 raters were then introduced to the task, with full instructions provided in Supplementary material S3.

The survey consisted of 24 randomized utterances drawn from the Cinderella story retelling task and took approximately 10–15 min to complete. In Part I, raters judged whether each utterance was agrammatic or not. Twelve of the 24 items were labeled agrammatic based on annotations provided by AphasiaBank, and the remaining twelve were not agrammatic. Among the agrammatic items, half were AI-generated synthetic data and half were produced by an actual person with aphasia. An example trial is illustrated in Supplementary Figure S3.

In Part II, raters were presented with the same 24 utterances and asked to judge whether each was produced by AI or by a real person with aphasia, given a snippet of context (c.f., Supplementary Figure S3). Of these items, 12 were AI-generated synthetic utterances and 12 were real, authentic productions from persons with aphasia.

All stimuli were randomized within their respective Parts. To increase statistical power, we replicated the survey design across four additional surveys. Across all five surveys, we collected ratings on 120 utterances in total from twelve raters. Each survey implemented an identical structure consisting of 24 items arranged in two blocks, as described earlier. Because raters were not required to evaluate the exact same utterance instances, reliability was assessed at the level of judgment types rather than individual items. Here, “types” refers to the categorical evaluation dimensions defined by the task (e.g., agrammatic vs. non-agrammatic; AI-generated vs. person with aphasia produced), which were held constant across surveys. Ratings were therefore aggregated by type across surveys. Inter-rater reliability was quantified using Fleiss’ Kappa, computed separately for each judgment type, and evaluated following Landis and Koch (1977). Confusion matrices were also reported to show how rater judgments aligned or diverged from the true labels, which had been pre-defined using AphasiaBank annotations prior to the survey.

For the online survey, we recognized that detecting agrammatic utterances from a single isolated utterance would not be reliable or interpretable in a clinically meaningful way. Moreover, without sufficient, aphasia-specific knowledge, such an utterance could also hinder the efficient learning of LLMs. On the other hand, including the entire discourse sample in a single rating or finetuning the models on the entire sample would be challenging due to a significant reduction in data points (there would be fewer agrammatic utterances since multiple would be in one sample). To address these concerns, we used a moving window strategy. This approach, previously applied in (clinical) NLP for addressing data scarcity and contextualizing limited data (Iter et al., 2018; Parola et al., 2023; Cong, 2024), allows models to better learn patterns by segmenting transcripts into smaller, contextualized utterances rather than longer paragraphs or discourse segments.

Concretely, our design was guided by the following rationale. Previous work in clinical NLP has applied the moving window strategy to incorporate discourse context, including its use in detecting incoherent speech in schizophrenia (Iter et al., 2018). From a computational perspective (Liu et al., 2024; Touvron et al., 2023; Grattafiori et al., 2024), model performance tends to be strongest when relevant information appears at the beginning or end of the input, but declines substantially when key details are embedded in the middle of longer contexts (so-called “lost in the middle”), even for models designed to handle extended inputs. In other words, while LLMs can process thousands of tokens, shorter, focused windows may help reduce the influence of “lost in the middle” and repetitive or off-target content, thereby maximizing the signal for the target line. Overall, we used a moving window strategy with a window size of five utterances, based on a pragmatic balance between contextual information and signal specificity. This window size corresponds to a typical narrative segment in our datasets and provides sufficient context (approximately 25–75 words) for identifying surface-level grammatical disfluencies while ensuring the target utterance remains the focal point for both human raters and LLMs. We additionally conducted exploratory comparisons with three-utterance context windows, to collect preliminary evidence of the extent to which our approach is robust to context window size.

We hypothesized that LLMs can generate synthetic data that captures surface-level features associated with agrammatic language. Accordingly, we expected higher inter-rater reliability in Part I, where raters judged the presence of agrammatic utterances, and lower reliability in Part II, where raters attempted to distinguish between AI-generated and human-produced utterances, reflecting the difficulty of differentiating sources when surface-level features overlap.

### 3.2 Study 2: evaluate language models with different training conditions

We evaluated language models under various training conditions, focusing primarily on pre-trained and fine-tuned LLMs, with a classic logistic regression model serving as an interpretive baseline model. Our goal was to examine how differences in training scope and architecture affect language modeling performance, particularly in the context of detecting agrammatic and surprisal-based utterances. Pre-trained LLMs are pre-trained on massive, diverse corpora using advanced architectures such as dense transformers or mixture of experts (MoE), enabling them to learn rich, contextual representations of language and exhibit broad linguistic capabilities. Fine-tuned LLMs were pre-trained LLMs that underwent further adaptation using aphasia speech transcripts. One of the purposes of fine-tuning is to evaluate how domain-specific data can shape and influence these models. In contrast, the logistic regression model does not undergo the LLM-style pre-training: it learns only from a small, domain-specific corpus (i.e., the aphasia datasets we compiled) and relies on relatively simple statistical techniques. By comparing these models, we aimed to assess both the limitations and the potential of different training strategies for modeling language impairments, and to explore how computational models might inform research in aphasia and psychological research more broadly.

It is worth emphasizing that with respect to the fine-tuned LLMs, the pre-trained LLMs serve as a vanilla baseline, representing what a general-purpose model knows without clinical adaptation. Fine-tuned LLMs are those same base models further adapted on aphasia-specific data. This comparison quantifies the added value of domain-specific fine-tuning. Both versions are evaluated on the same held-out real test set, ensuring valid comparison.

#### 3.2.1 Task 1: agrammatic utterances detection

We first evaluated pre-trained LLMs’ performance in classifying the presence of agrammatic utterances in the real dataset, aiming to explore *pre-trained* LLMs’ clinical efficacy. The task involved predicting whether a given utterance is agrammatic. Second, we fine-tuned LLMs using the augmented dataset which involved both real and synthetic agrammatic data. By *augmented dataset* in this paper, we mean the combination of the real datasets and synthetic datasets. We then evaluated the extent to which such fine-tuning improved LLMs’ performance.

We used the same moving window strategy as illustrated in the human surveys. All human raters’ as well as LLMs’ inputs were structured in the same format to reinforce ratings’ consistency and models’ learning, hence ensuring valid comparisons. The window size was five, for both human surveys and LLMs, meaning for each utterance, we created a snippet of five utterances to facilitate LLMs’ learning as well human clinicians’ rating. In example (1), we illustrated how we contextualized each utterance in a window size of five (c.f. Supplementary material S2 for more details and additional information about a window size of three):

(1) <patient_story>
   *and starts to rain* [line 1; label: 0]
   *and when he gets back home he is wet* [line 2; label: 0]
   *mother not happy* [target line; label: 1]
   *so I guess he’s made a decision the next time he’ll take the umbrella with him as it is raining* [line 4; label: 0]
   *well I guess that a* [line 5; label: 0]
   </patient_story>
   <target_line>*mother not happy*</target_line>


As shown in (1), a label of 1 represents the presence of agrammatic utterances and 0 grammatical speech, namely the absence of agrammatic utterances. LLMs were fine-tuned to predict the agrammatism label in the target line. To ensure fair comparison and effective learning, we used all the lines with label 1 first, and we randomly sampled an equal number of lines with label 0. In edge cases, for example, if line 1 happens to be the target utterance, we take this line and the next four lines as a snippet. If the last line happens to be the target utterance, we take all four lines before it to create a snippet. Each snippet always consisted of five lines of utterances produced by a person with aphasia in a given task.

We used Llama-3.1-8b-instruct (Grattafiori et al., 2024), an auto-regressive language model with optimized transformer architecture and about eight billion parameters. To strengthen our pipeline and validate our examination in a separate LLM, we additionally included Gemini Flash 2.0 (Google, 2025), also an auto-regressive model, which has a large input token window that is comparably appropriate for sufficient contextualization. Both LLMs generate text through making predictions about the next word in a sequence, based on the previous text it has generated. These two LLMs were chosen to examine how differences in model properties may influence task performance. We speculate that dense transformer models like Llama may be better suited for capturing fine-grained linguistic dependencies relevant to detecting agrammatic features and language dysfunction more broadly (Grattafiori et al., 2024), while Gemini Flash 2.0’s MoE architecture and extended context window may facilitate integration of broader discourse-level cues (Google, 2025), but we emphasize that the current design cannot isolate architectural factor. These model differences reflect not only technical distinctions but may also influence task performance, including the ability to capture patterns of language atypicality central to neuropsychological and cognitive-linguistic assessment.

Data were balanced and split for training and testing. The training–testing split ratio is 0.9. For the real data, there were 672 snippets in total for the training set, and 74 snippets in the testing set (37 for each label: presence and absence of agrammatic utterances). For the AI-generated synthetic data, there were 5,400 snippets in training and 600 in testing (300 for each label: presence and absence of agrammatic utterances). We used the same datasets to fine-tune both LLMs to ensure valid comparisons across models. Due to LLM model accessibility differences, different fine-tuning mechanisms were adapted for each LLM model: Parameter Efficient Fine-Tuning (PEFT) with LoRA (Low-Rank Adaptation, Hu et al., 2022) for the open-sourced Llama 3.1-8b-instruct, which substantially reduces the trainable parameters while preserving the model’s linguistic capabilities, and Supervised Fine-Tuning for the close-sourced Gemini Flash 2.0. We limited fine-tuning to one epoch due to the small dataset size, the rapid adaptation capability of LLMs, and to mitigate potential overfitting.

To establish a baseline with a classic machine learning model, we used both the real (human produced) and AI-generated datasets to train and evaluate a logistic regression model. To mirror the experiments which were carried out using LLMs as evaluative models, we used a feature engineering technique that extracted the target line and appended it along with the patient story to create a combined input, which will be operated on to create features for training. We used a combination of bigrams (two-word sequences) and TF-IDF (Term Frequency-Inverse Document Frequency) to create these features. Concretely, bigrams capture the broken transitions and missing function words typical of agrammatic speech. TF-IDF downweighs common, uninformative words while boosting rare, agrammatism-specific patterns, likely allowing a simple linear model to achieve strong discriminative power for surface-transparent linguistic signals.

Since agrammatic speech contains unusual missing words and incomplete sentence constructions, the rarity of these patterns further emphasizes the usage of TF-IDF in boosting inputs that contain these rare patterns. Furthermore, it helps in downweighing the contribution of redundant content that may occur across all training samples. Additionally, bigrams ensure such broken patterns are captured more frequently. Moreover, including the target line twice (once in the patient story and once in the target line tag) serves as a naive attention mechanism to ensure bigrams of this target line are given more weight by the model while making predictions. This feature creation method was applied to training both versions of the logistic regression model, one that was trained and tested on real data, and the other that was trained on augmented data and tested on AI-generated synthetic data only.

Performance was evaluated using standard classification metrics such as precision, recall, and F1 Score, and was visualized with confusion matrices to analyze per-class metrics. For Task 1 (agrammatic utterance detection), the reliability of these comparisons is constrained by a limited real-data test set (*n* = 74), which precluded formal significance testing. In contrast, Task 2 involved larger datasets, enabling more systematic significance testing and additional experiments, as described next.

#### 3.2.2 Task 2: detection of LLM-surprisals indexing linguistic atypicality

We applied the same pipeline used in classifying agrammatic utterances in Task 1 to the detection of LLM-surprisal based utterances. The same moving window strategy, with a window size of five, was applied. The same two LLMs, fine-tuning settings, and data splitting and balancing strategies described for agrammatic utterances were also used in this task. For the real data, after balancing and data splitting, there were 5,400 snippets in the training dataset and 600 such data points in the testing dataset. As to the synthetic LLM-surprisal indexed data, after balancing and splitting, there were 7,200 snippets in the training dataset and 800 such data points in the testing dataset.

Due to the availability of larger data size for Task 2 compared to Task 1, we were able to conduct more systematic experiments for Task 2. The methodology here involved using different training paradigms across multiple instances of the same model and evaluating and comparing their performances on a real (human produced) LLM-surprisal indexed dataset. Our goal for each LLM was to have three evaluation platforms. The first one involved using a pre-trained LLM as a base. This was followed by fine-tuning another instance of the same LLM on the real, human-generated LLM-surprisal indexed dataset. Finally, we fine-tuned a third instance of the LLM on an augmented dataset, which also included AI-generated synthetic LLM-surprisal data. The test dataset, which contained real LLM-surprisal indexed data, was kept the same across all three cases to enable valid comparisons.

Furthermore, to establish a baseline with a classic machine learning model, we trained two instances of the logistic regression model on the real and the augmented utterances, respectively, and evaluated them on real utterances. The same feature engineering pipeline used in the agrammatic utterance detection task (Task 1) was applied to Task 2. The same fine-tuning settings and evaluation metrics used in Task 1 were applied to Task 2.

Following this methodology would enable us to answer three sub-questions: Does fine-tuning help within a given LLM? Does AI-generated synthetic LLM-surprisal data improve LLMs’ performance on real data? and Do LLMs add value over classical machine learning models in detecting LLM-surprisal indices derived from real aphasic utterances?

To statistically validate the differences within-LLMs and between LLMs and the baseline logistic regression models that were trained using different training regimes, we used the McNemar’s test to compare model performances on real aphasic utterances. This enabled us to deliver robust conclusions with respect to the inferences corresponding to the LLM-surprisal indexed utterance detection task. McNemar’s Tests were only applied to Task 2, because sample sizes in Task 1 were relatively small to meaningfully apply the McNemar’s tests.

### 3.3 Study 3: exploratory analyses of aphasia severity prediction

To address the need for more clinically fine-grained subgroup analyses and evaluate the scalability of LLM-based clinical staging, we conducted an exploratory analysis predicting graded aphasia severity from spontaneous speech transcripts. Extending beyond Study 2’s binary classification approach (e.g., agrammatic vs. non-agrammatic), Study 3 evaluated whether a relatively lightweight LLM (Llama-3.1-1B, Grattafiori et al., 2024) could handle *multi-class* classification distinguishing among four clinically recognized levels of aphasia severity at the utterance level, given the surrounding discourse context produced by actual PWA.

Severity labels were derived from Western Aphasia Battery-Revised Aphasia Quotient (WAB-R AQ, Kertesz, 2006) scores using established clinical thresholds: severe (AQ 0–50), moderate (AQ 51–75), mild (AQ 76–93.8), and latent (AQ > 93.8, which reflects WAB-R performance above the aphasia cutoff, although (subtle) impairments may still be evident in daily communication). These AQ scores were already available and provided in the AphasiaBank.

We selected the WAB-R AQ over the WAB-R spontaneous speech subscores (fluency and information content) for two main reasons. First, the spontaneous speech subscores rely on 10-point rating scales that are relatively granular for an initial proof-of-concept multi-class analysis. Second, and more importantly for the primary aim of Study 3, which was to evaluate graded severity classification rather than fluent versus nonfluent speech distinctions, the WAB-R AQ severity categories provide clinically established intermediate groupings that are better suited for evaluating whether an LLM can distinguish among multiple levels of aphasia severity. Additionally, the AQ captures impairment across several language domains, providing a broader measure of overall aphasia severity beyond connected speech performance alone, making it more appropriate for this exploratory extension of Study 2.

Although the WAB-R fluency subscore more directly reflects connected speech production, fluent versus nonfluent distinctions are fundamentally binary, and the 10-point fluency scale is relatively granular for multi-class analysis. To demonstrate computational feasibility and to show that how our approach can generalize to various WAB subdomain scores, we conducted a supplementary analysis on a subset of pipelines and models, assessing whether WAB-R fluency severity (fluent: 5–10 vs. nonfluent: 0–4; Kertesz, 2006; Clough and Gordon, 2020) is detectable from utterance-level text alone. Our report focused on WAB-R AQ, with WAB-fluency results provided in the Supplementary materials.

We obtained 482 PWA transcripts from the AphasiaBank (283 male, 199 female; mean age 61.3). The transcripts spanned multiple discourse tasks, as defined by the AphasiaBank “Task” configuration, including personal narratives, picture descriptions, story retelling, and procedural discourse (https://talkbank.org/aphasia/; Macwhinney and Fromm, 2023; MacWhinney et al., 2011). For this exploratory analysis, we did not restrict the dataset to Cinderella story retellings and picture descriptions, unlike the main analyses. This decision allowed us to (a) construct an independent dataset for evaluating a different model, (b) assess the validity and generalizability of findings from the primary analyses, and (c) intentionally vary discourse topics (i.e., “tasks” as coded in the AphasiaBank), which served as manipulated input context in our LLM prompting ablation experiments.

Specifically, we created pairs with each conversation’s associated discourse topic. Each pair was formatted as “{topic}: {utterance},” where {topic} referred to “Task” as configured in the AphasiaBank, to provide Llama-3.1-1B with contextual information about the topic and the subject matter of the PWA’s speech, and the input text included both the surrounding discourse paragraph (context) and the target utterance. This formatted input was nested within the instruction prompt presented to Llama-3.1-1B. The model was explicitly instructed to predict an aphasia severity label for the target utterance only, while using the broader discourse context to inform its judgment. We illustrated an example as follows:

(2) *A sample prompt in LLM severity detection (for illustration purposes):*
	*You are a speech classifier. Your job is to assess the severity of a patient’s aphasia based on a given utterance and the topic of the utterance.*
	*The input will be formatted as “topic:utterance.”*
	*If you see that the speech is unrelated to the topic, it is likely the patient has a stronger aphasia. Put each message into one of four categories: latent/mild/moderate/severe. Only respond with the category.*
	*Here are some examples:*
	*Text: Cinderella: uh uh there was where his wife gir:l. uh: and we caused her her. She she she could … [the whole discourse of Cinderella story retelling].*
	*Category: Severe [the same format for other categories].*
	*Here is the one you need to classify:*
	*Text: {topic}: {utterance}*
	*Category*:

We tested each pair on four types of prompts to examine the effects of prompt ablation type on model performance: (1) short prompt: a direct instruction to classify severity; (2) pattern recognition: providing structural cues about aphasic speech patterns; (3) few-shot: providing examples of each severity level within the context window; (4) few-shot + Chain of Thought (CoT): encouraging the model to reason through linguistic features (e.g., agrammatic, fluency) before classifying. All the instructions, patterns and features explanations in the prompts were sourced from the AphasiaBank (https://talkbank.org/aphasia/; https://talkbank.org/aphasia/examples/index.html; Macwhinney and Fromm, 2023; MacWhinney et al., 2011). Detailed prompts for the other prompting ablation types are provided in Supplementary material S4 and the OSF repository.

We parsed LLM’s responses with standardized computer programs. LLM tended to add filler words, reported multiple categorizations, as well as other (irrelevant) details. To parse the categorization most reported by the LLM, we took the severity with the highest frequency as the LLM’s final response. Given four severity classes, chance performance is approximately 25%. We reported classification reports in addition to confusion matrices for describing breakdown performances.

## 4 Results

### 4.1 Study 1: findings—AI-generated synthetic data validation

In the offline survey, the two raters identified 81% of the 3,000 synthetic data points as agrammatic utterances, and they reported a confidence rating of 5 (very confident) for over 96% (2,995 utterances), providing preliminary evidence that the LLM outputs were generally consistent with the prompting criteria derived from the AphasiaBank.

**Figure 1:**
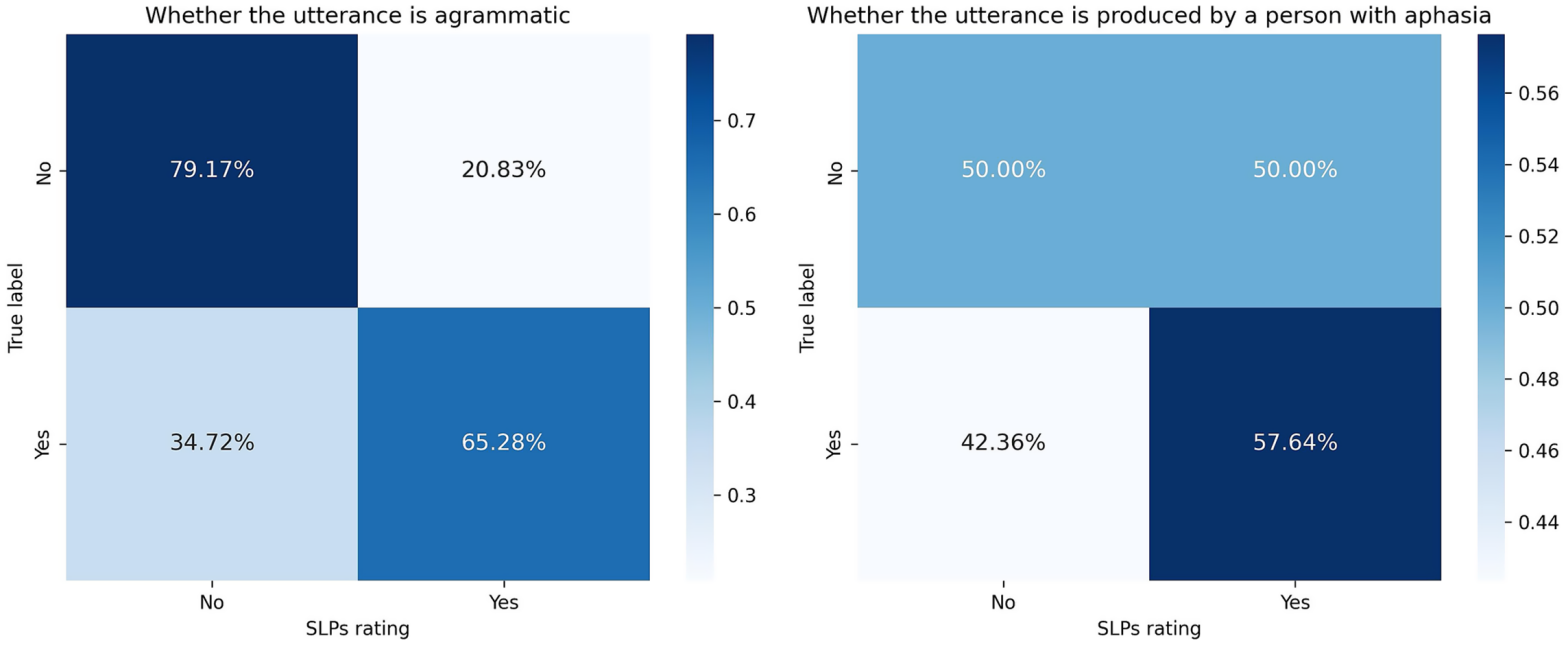
Confusion matrices for parts 1 (left) and 2 (right) of the web-based survey. Darker diagonals indicate a higher alignment between true labels and human participants’ ratings, suggesting that more aphasia-like characteristics were captured, hence better data quality.

In the web-based human evaluation survey, Figure 1 illustrates SLP raters’ and trained RAs’ responses to Parts 1 and 2 of the survey, which serves as our primary test of this hypothesis. In Part 1, where participants judged whether an utterance was agrammatic or not, 65.28% of “Yes” responses aligned with the true label (agrammatic), while 34.72% of the time raters responded “No” to utterances that were actually agrammatic. Conversely, for the non-agrammatic utterances, the raters indicated “No” 79.17% of the time when an utterance was not agrammatic, but they judged 20.83% of the non-agrammatic utterances as agrammatic. These results indicated that the AI-generated synthetic data showed above chance level resemblance to real aphasia data. Further, the relatively higher alignment for non-agrammatic utterances suggested that the AI-generated grammatical utterances more successfully mimicked non-agrammatic speech, whereas the synthetic agrammatic utterances were less reliably perceived as being agrammatic, potentially reflecting the inherent complexity of simulating authentic agrammatic utterances.

In Part 2, which asked whether an utterance was produced by a real person with aphasia, 57.64% of “Yes” responses were associated with utterances produced by a real person, while 42.36% of the time the raters responded “No” to utterances that were in fact produced by a person with aphasia. For utterances generated by AI, raters correctly chose “No” 50% of the time, and were wrong 50% of the time, believing those AI-generated utterances were from real individuals with aphasia. This at or below-chance performance suggests that raters were often unable to distinguish between AI-generated and human-produced utterances. Taken together, these findings imply that while raters could somewhat reliably detect grammatical impairments, they were less consistent when judging source differences. Yet, these results preliminarily suggest that AI-generated data may capture certain surface-level features associated with agrammatic language, laying the foundation for future clinical fidelity studies.

The Fleiss’ Kappa for inter-rater agreement across all the trials was 0.18, indicating slight agreement among the 12 raters. The Fleiss’ Kappa for Part 1 was 0.328, suggesting fair agreement. The Fleiss’ Kappa for Part 2 was −0.03, suggesting that the raters’ agreement was less than what would be expected by chance. In other words, raters disagreed more than would be expected by random guessing. Since raters could not confidently tease apart the synthetic from the human-produced speech, it may reflect task-related factors, including the inherent difficulty of the classification task and the limited clinical context provided by the five-utterance snippets. Alternatively, this pattern may suggest that AI-generated utterances capture some surface-level features associated with agrammatic language. We discuss these implications in-depth in the Discussion section, and we leave full validation of this proposed explanation for future studies.

Rater heterogeneity (SLPs vs. student RAs) might be a potential confound. While all raters confirmed proficiency and were trained using the same AphasiaBank criteria, we recognize that their clinical experience varied. Therefore, we conducted a subgroup *t*-test analysis, comparing the mean match rates for Part 1 and Part 2 between student RAs (self-report 0 years of clinical experience working with individuals with aphasia, *n* = 6) and non-student raters (self-report at least 0.5 years of clinical experience working with individuals with aphasia, *n* = 6). No statistically significant difference was found between the groups: Part 1 student RAs Mean = 0.646, SD = 0.08; Non-Student raters Mean = 0.587, SD = 0.074; *t* = 1.327, *p* = 0.214 (Bonferroni adjusted *p* = 0.428). Part 2 Student RAs Mean = 0.465, SD = 0.037; Non-Student raters Mean = 0.51, SD = 0.034; *t* = −2.21, *p* = 0.052 (Bonferroni adjusted *p* = 0.103).

In summary, we provided a preliminary proof-of-concept that AI-generated agrammatic speech captures some surface-level features associated with agrammatic language, and that LLMs have the potential to model, classify, and generate similar surface-level patterns.

### 4.2 Study 2: findings—detecting agrammatic language and LLM-surprisals indexing linguistic atypicality

#### 4.2.1 The overall performance

Table 1 showed the macro-F1 scores and corresponding 95% bootstrapped Confidence Intervals (CI) for Task 1 (agrammatic utterance detection). McNemar test results on real, human-produced data showed that the baseline logistic model outperformed both Llama and Gemini in detecting agrammatic utterances. We did not find solid evidence to conclude that Llama and Gemini were significantly different from each other. For AI-generated synthetic data, the baseline model outperformed Llama and Gemini both again, whereas Llama significantly outperforms Gemini.

**Table 1:** Models’ performance in classifying the presence of agrammatic utterances.

Evaluation dataset	Training dataset	Baseline—logistic regression model	Llama	Gemini
Real data	Real data for the baseline model; N.A. for pretrained LLMs	0.689 [0.579, 0.794]	0.41 [0.295,0.692]	0.386 [0.27,0.495]
Synthetic data	Augmented data	**0.758 [0.723, 0.79]**	0.653 [0.614,0.692]	0.508 [0.468,0.548]

Values represent F1 Score and bootstrapped CIs. N.A.: not applicable, since LLM is already pretrained and its general-purpose pretraining did not involve training on the real data. The best-performance model is highlighted in bold.

For the additional experiment on Task 1 with three-utterance context window, regarding the baseline logistic regression model, when trained and tested on real data, results showed that bootstrapped F1 = 0.716, 95% CI = [0.608, 0.811]; when trained and tested on synthetic data, the bootstrapped F1 = 0.788, 95% CI = [0.754, 0.82]. This slight improvement in overall performance suggests that, for the linear machine learning model, a shorter context window was associated with better overall results, likely because it increased the sample size and provided more data points for the model to learn from due to smaller text segmentations.

For Task 2, Table 2 showed the macro-F1 scores and corresponding 95% bootstrapped CIs for the performance of Llama and Gemini model instances in classifying real, human-generated LLM-surprisals indexing linguistically atypical utterances, categorized by training mechanisms. From these metrics alone, we observe an improvement in the F1 Score when LLMs were fine-tuned with the LLM-surprisal index data, as compared to the pre-trained versions from Task 1. To examine this observation, we conducted McNemar’s test on each LLM model and its instances, with the Bonferroni correction (alpha = 0.05/3 = 0.0167) for valid and simultaneous comparisons applied.

**Table 2:** Models’ performance in classifying the presence of LLM-surprisal indexed atypical, aphasic-like utterances.

Evaluation dataset	Training dataset	Baseline—logistic regression model	Llama	Gemini
Real data	Pre-trained	N.A.	0.519 [0.48, 0.561]	0.386 [0.349, 0.423]
Real data	Real data	0.668 [0.63, 0.706]	0.856 [0.829, 0.885]	0.752 [0.718, 0.785]
Real data	Augmented data	0.701 [0.663, 0.737]	**0.887 [0.862, 0.912]**	0.745 [0.709, 0.78]

Values represent F1 Score and bootstrapped CIs. N.A.: not applicable, since logistic regression is not a pre-trained model as in LLM pre-training sense. The best-performance model is highlighted in bold.

For the additional experiment on Task 2 with three-utterance context window, regarding the Llama model, when fine-tuned using real data and evaluated on real test data, bootstrapped F1 = 0.691, 95% CI = [0.655, 0.725]; when fine-tuned using augmented data and evaluated on real test data, bootstrapped F1 = 0.743, 95% CI = [0.708, 0.778]. This decrease in overall performance suggests that for Llama, which excels at long-context comprehension and generation, the negative impact of reducing the context window outweighed the advantage of having more data points from smaller text segmentations. On the other hand, regarding the baseline logistic regression model, when trained and tested on real data, bootstrapped F1 = 0.701, 95% CI = [0.663, 0.737]; when trained on augmented data and tested on real data, bootstrapped F1 = 0.739, 95% CI = [0.702, 0.773]. This finding resonates with the logistic regression model’s performance in Task 1, suggesting that for linear machine learning models, regardless of tasks, the advantage of having more data points from smaller text segmentations outweighed the negative impact of reducing the context window.

Table 3 showed that for Task 2 (classification based on the surprisal-derived index of linguistic atypicality), fine-tuning the Llama model leads to significant improvements in predictions when compared with the pre-trained Llama model. This can be observed from the test statistic after applying the Bonferroni correction. However, even though directionality slightly trends towards an improvement after augmentation, its impact is not significant, suggesting that differences between Llama fine-tuned on just the real data and Llama fine-tuned on augmented data are minimal, resonating with findings in Table 2.

**Table 3:** McNemar’s test comparisons for Llama 3.1-8b-instruct in classifying the presence of LLM-surprisal indexed atypical, aphasic-like utterances.

Comparison (model_1 vs. model_2)	Number of instances where (model_1 correct, model_2 wrong)	Number of instances where (model_1 wrong, model_2 correct)	McNemar’s test statistic	*p*-value
**Pre-trained vs. Fine-tuned on real data**	**31**	**220**	**140.813**	**<0.0001**
**Pre-trained vs. Fine-tuned on augmented data**	**34**	**241**	**154.313**	**<0.0001**
Fine-tuned on real data vs. Fine-tuned on augmented data	21	39	4.817	0.028

Significant comparisons are highlighted in bold.

Similarly, Table 4 showed that for Task 2 (classification based on the surprisal-derived index of linguistic atypicality), fine-tuning Gemini produced a significant improvement in predictions when compared with the base pre-trained model. However, fine-tuning Gemini with augmented data does not introduce any significant improvement in predictions as compared to the Gemini model fine-tuned on LLM-surprisal indexed aphasic utterances derived from real human data. These findings align with what we observed from the reported F1 scores in Table 2.

**Table 4:** McNemar’s test comparisons for Gemini flash 2.0 in classifying the presence of LLM-surprisal indexed atypical, aphasic-like utterances.

Comparison (model_1 vs. model_2)	Number of instances where (model_1 correct, model_2 wrong)	Number of instances where (model_1 wrong, model_2 correct)	McNemar’s Test Statistic	*p*-value
**Pre-trained vs. Fine-tuned on real data**	**63**	**259**	**118.090**	**<0.0001**
**Pre-trained vs. Fine-tuned on augmented data**	**56**	**249**	**120.866**	**<0.0001**
Fine-tuned on real data vs. Fine-tuned on augmented data	91	88	0.022	0.881

Significant comparisons are highlighted in bold.

To examine whether fine-tuned LLMs outperform the logistic regression baseline model in Task 2 (classification of LLM-surprisal index), we conducted McNemar’s tests on paired predictions, comparing each LLM model instance for a particular training condition to the corresponding logistic regression model. Since four planned comparisons were tested, we applied the Bonferroni correction to adjust the significance level. Results of these comparisons are shown in Table 5.

**Table 5:** McNemar comparisons (LLMs vs. logistic regression) for training using real data in LLM-surprisal indexed atypical, aphasic-like utterance classification.

Comparison (training mechanism) (model_1 vs. model_2)	Number of instances where (model_1 correct, model_2 wrong)	Number of instances where (model_1 wrong, model_2 correct)	McNemar’s test statistic	*p*-value
**Llama vs. Logistic regression (real data)**	**152**	**39**	**65.675**	**<0.0001**
**Llama vs. Logistic regression (augmented data)**	**147**	**37**	**64.571**	**<0.0001**
**Gemini vs. Logistic regression (real data)**	**132**	**82**	**11.219**	**0.0008**
Gemini vs. Logistic Regression (augmented data)	122	96	2.867	0.0904

Significant comparisons are highlighted in bold.

Table 5 showed that for Task 2 (classification of LLM-surprisal indexed aphasic utterances derived from real human data), in all cases, LLMs correctly classify more examples than the logistic regression model and in three out of four cases, this difference is statistically significant. Even in the fourth case, the LLM identifies more errors than the logistic regression model, but this metric is not significant. This analysis suggests that LLMs may provide advantages over a logistic regression baseline on the surprisal-based classification task, which uses surprisal as a derived index of linguistic atypicality.

#### 4.2.2 The breakdown of performance

**Figure 2:**
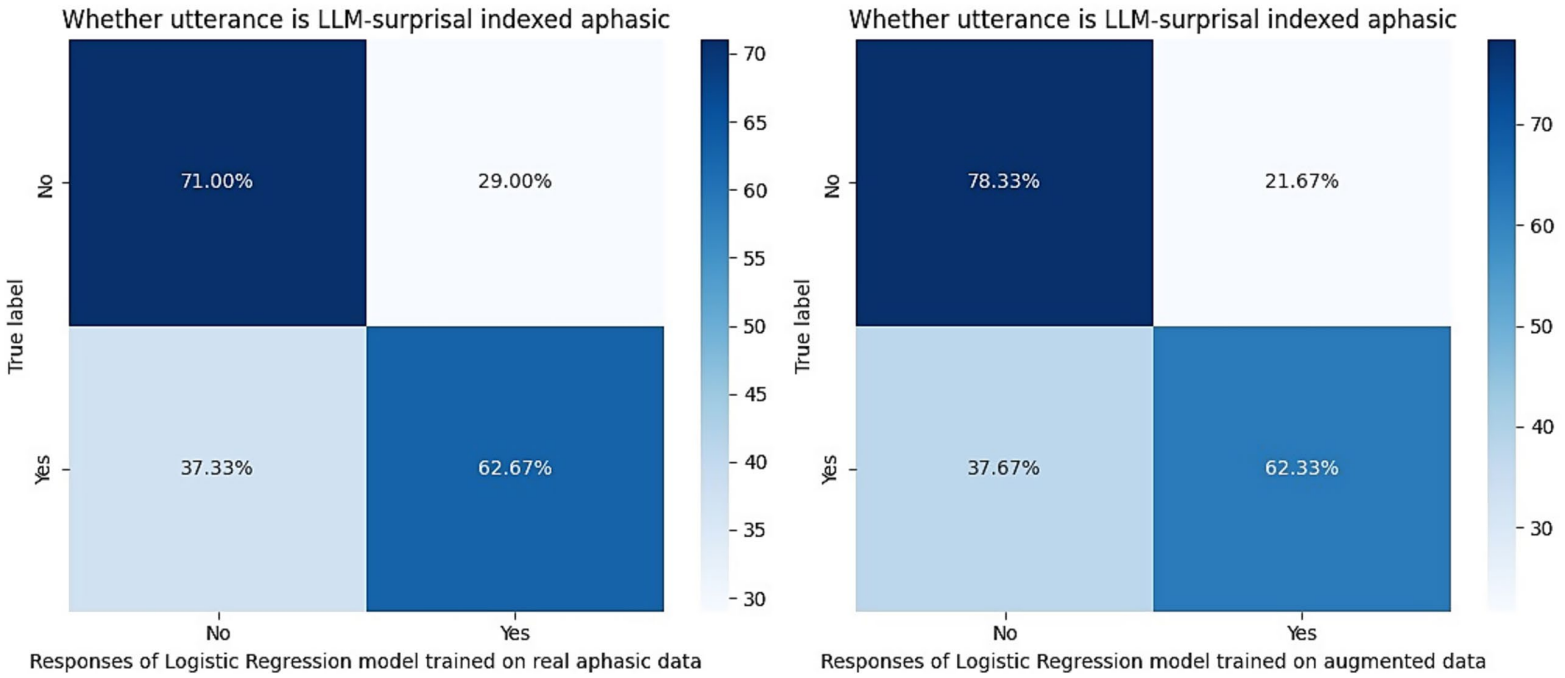
Confusion matrices of the logistic regression model trained on real (left) and the logistic regression model trained on augmented LLM-surprisal indexed atypical, aphasic-like data (right).

For Task 2, the classification of the LLM-surprisal index, we additionally provided breakdown of model performance, since it involved larger datasets and more systematic modeling. Figure 2 shows performance of two logistic regression models, one is trained using LLM-surprisal indexed aphasic data produced by real persons with aphasia, and the other is trained using augmented data. Both are evaluated on real LLM-surprisal data. We use this as a baseline to compare against LLMs fine-tuned using the same training datasets.

**Figure 3:**
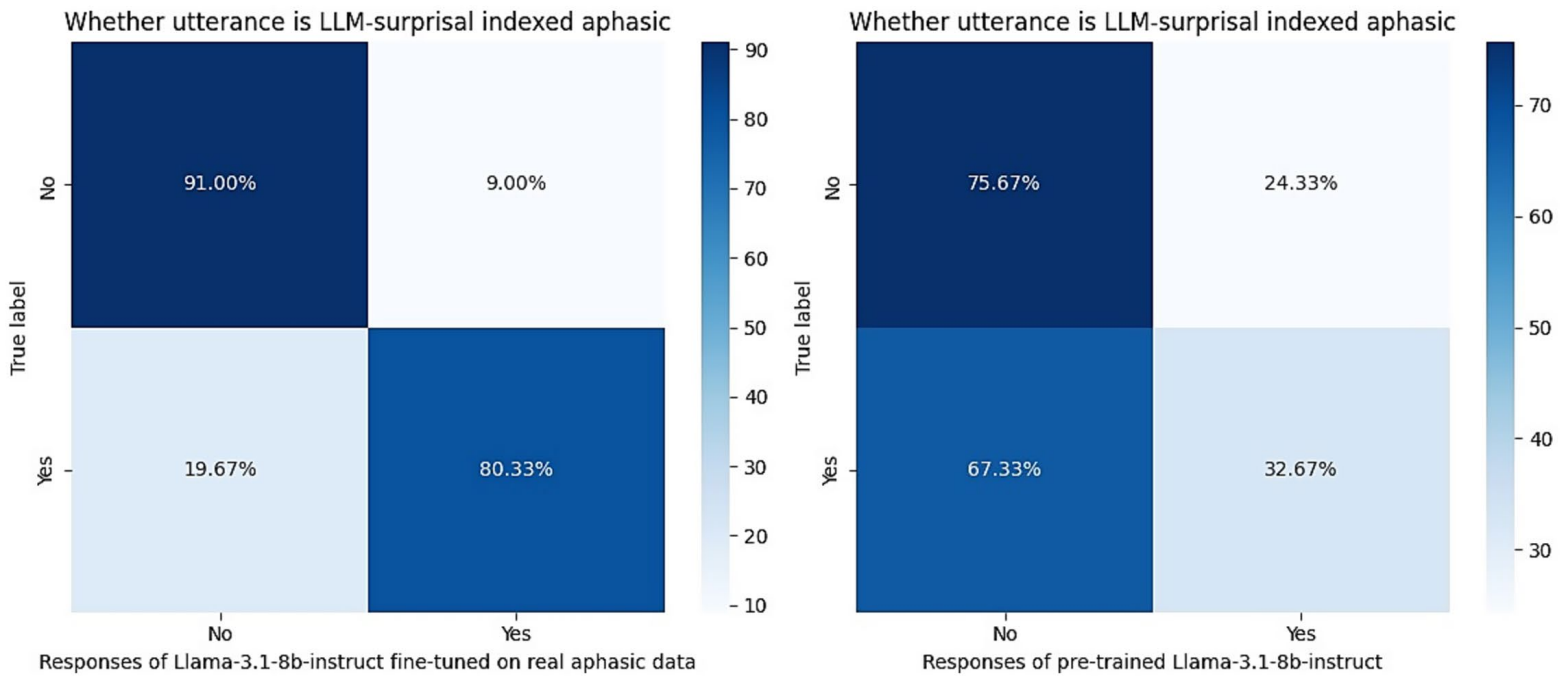
Confusion matrices of Llama fine-tuned using real LLM-surprisal indexed aphasic data (left) and pre-trained Llama (right) in classifying LLM-surprisal indexed atypical, aphasic-like utterances.

Figure 3 shows performance of two versions of Llama—the pre-trained model and the version fine-tuned on real data—in classifying utterances based on the surprisal-derived index of linguistic atypicality. Results suggest that fine-tuned Llama outperforms the pre-trained version in correctly detecting aphasic utterances (True positive (TP) rate of fine-tuned Llama = 80%, TP rate of pre-trained Llama = 33%) and correctly detecting non-aphasic utterances (fine-tuned Llama True Negatives (TN) rate = 91%, pre-trained Llama TN rate = 76%). Comparatively, pre-trained Llama also flagged more non-aphasic utterances as aphasic (24% False Positives (FP) as compared to Llama’s 9%).

It is worth noting the performance difference in Task 2 between pre-trained Llama (Figure 3, TP rate = 32.67%) and the logistic regression baseline models (Figure 2, TP rate = 62.67%) highlight limitations of general-purpose models in capturing task-relevant atypical, aphasic-like language patterns. While Llama is trained on vast corpora, this exposure primarily reflects “typical” language patterns, and may not provide the categorical structure needed to identify atypical, aphasic-like patterns without task-specific alignment. This interpretation is supported by Llama’s substantial performance gains following domain-specific fine-tuning (Figure 3, TP rate = 80.33%; Table 2, F1 = 0.856), suggesting that LLMs can learn task-relevant atypical, aphasic-like language patterns, but require explicit task-specific supervision to do so.

**Figure 4:**
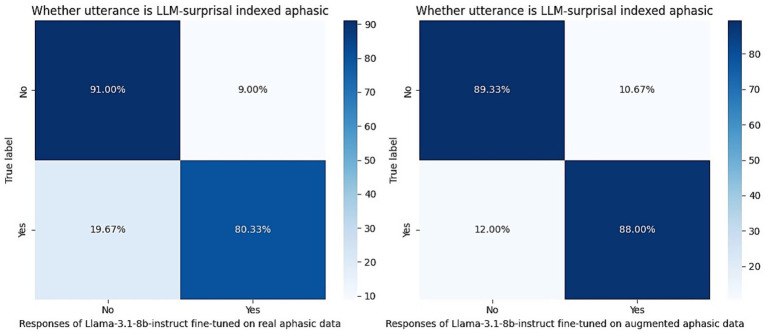
Confusion matrices for real data fine-tuned Llama (left) and augmented data fine-tuned Llama (right) in classifying LLM-surprisal indexed atypical, aphasic-like utterances.

Figure 4 shows performance of two versions of Llama in classifying utterances based on a surprisal-derived index of linguistic atypicality: one fine-tuned on real, human-generated surprisal data only, and the other fine-tuned on an augmented dataset that includes AI-generated synthetic data. We observe that after adding synthetic data into the fine-tuning mechanism, the model improved at detecting LLM-surprisal indexed utterances (8% improvement in TP rate for augmented model). Although this was complemented by a slight increase in FPs. This suggests that data augmentation improves classifier performance on the surprisal-based classification task, which indexes linguistically atypical, aphasic-like utterances.

**Figure 5:**
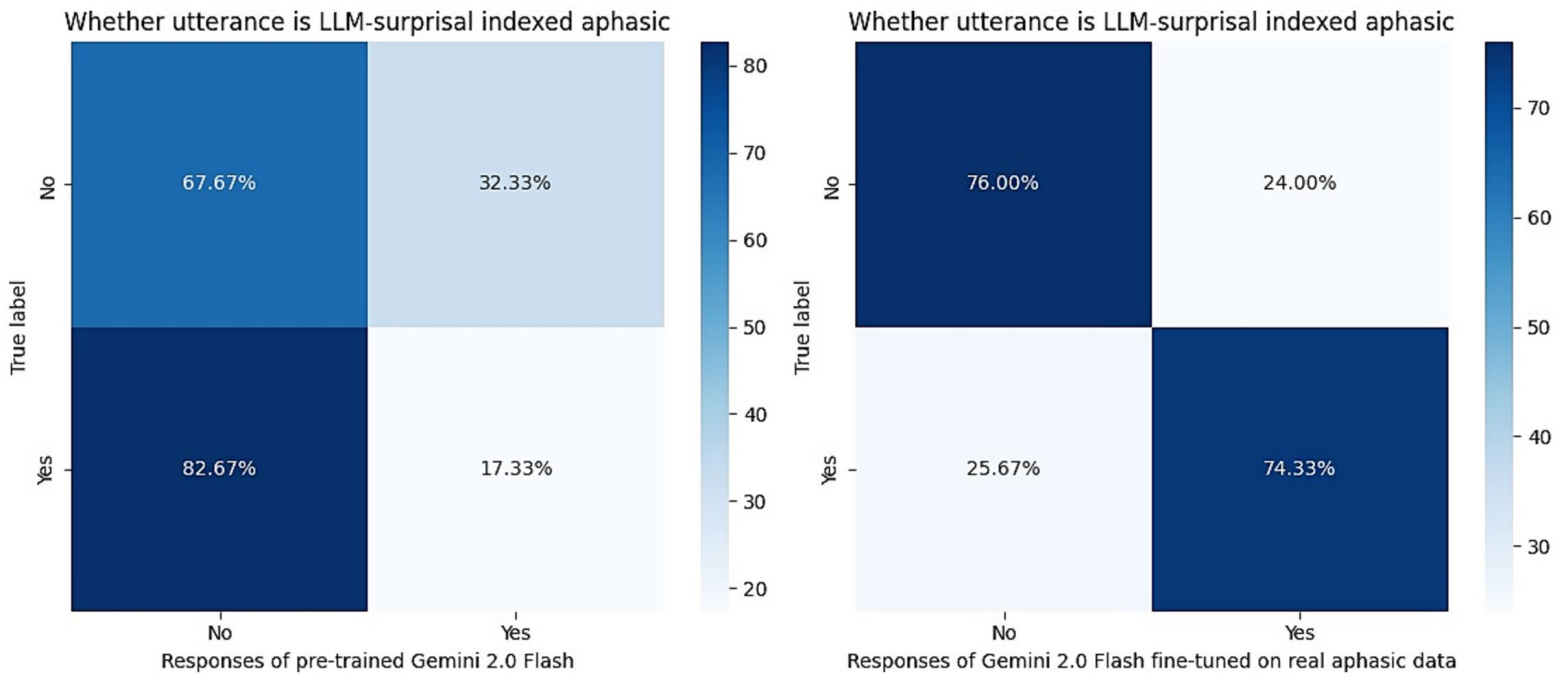
Confusion matrices for pre-trained Gemini model (left) and Gemini fine-tuned on real data (right) in classifying LLM-surprisal indexed atypical, aphasic-like utterances.

Figure 5 shows the performance of pre-trained Gemini and Gemini fine-tuned using real human-generated data. In line with what we observed in the Llama model comparisons, fine-tuning leads to a large increase in the model’s capability in correctly detecting LLM-surprisal indexed utterances (17.33 to 74.33%), with a similar decrease observed in False Negatives (82 to 26%).

**Figure 6:**
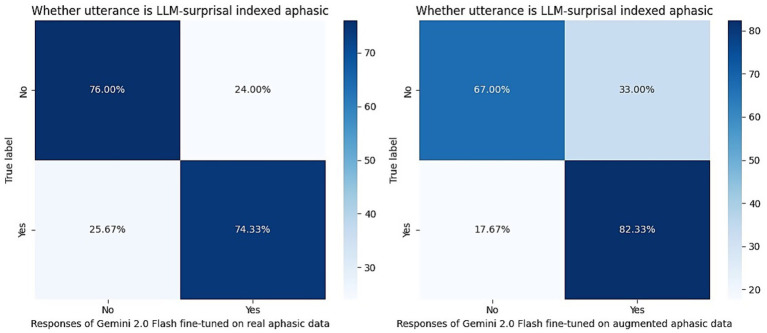
Confusion matrices for Gemini Flash 2.0 fine-tuned on real data (left) vs. fine-tuned on augmented data (right) in classifying LLM-surprisal indexed atypical, aphasic-like utterances.

Figure 6 shows the comparison of Gemini Flash 2.0 fine-tuned on real data versus the model fine-tuned on augmented data. We observe that the augmented model performs better in correctly classifying LLM-surprisal indexed utterances (an 8% increase in True Positives), but it also misclassifies more utterances as atypical, aphasic when they were actually typical, non-aphasic (9% increase in False Positives).

Taken together, results suggested that fine-tuning improves LLM performance on the surprisal-based classification task, with gains observed in correctly classifying both high (aphasic-like) and low (nonaphasic-like) surprisal utterances relative to pre-trained models. We proposed an approach for fine-tuning LLMs for language atypicality classification tasks. Our findings showed that performance varied from model to model, likely reflecting differences in a combination of many factors, for instance, model properties, and fine-tuning regimes used for Llama and Gemini due to accessibility constraints. For Llama, F1 scores on the surprisal-based task varied from 0.519 to 0.887. For Gemini, they ranged from 0.386 to 0.745. Moreover, text-only synthetic data augmentation was feasible, holding promise for generalization in future research. LLMs, particularly Llama, showed improved performance with fine-tuning and data augmentation, and LLMs outperformed the logistic regression model more often than not in Task 2. Overall, these results highlight the potential of text-based LLMs for modeling patterns of language atypicality in a preliminary setting, with implications for future research in aphasia and related clinical domains.

### 4.3 Study 3: findings—explore LLMs in end-to-end aphasia severity prediction

**Figure 7:**
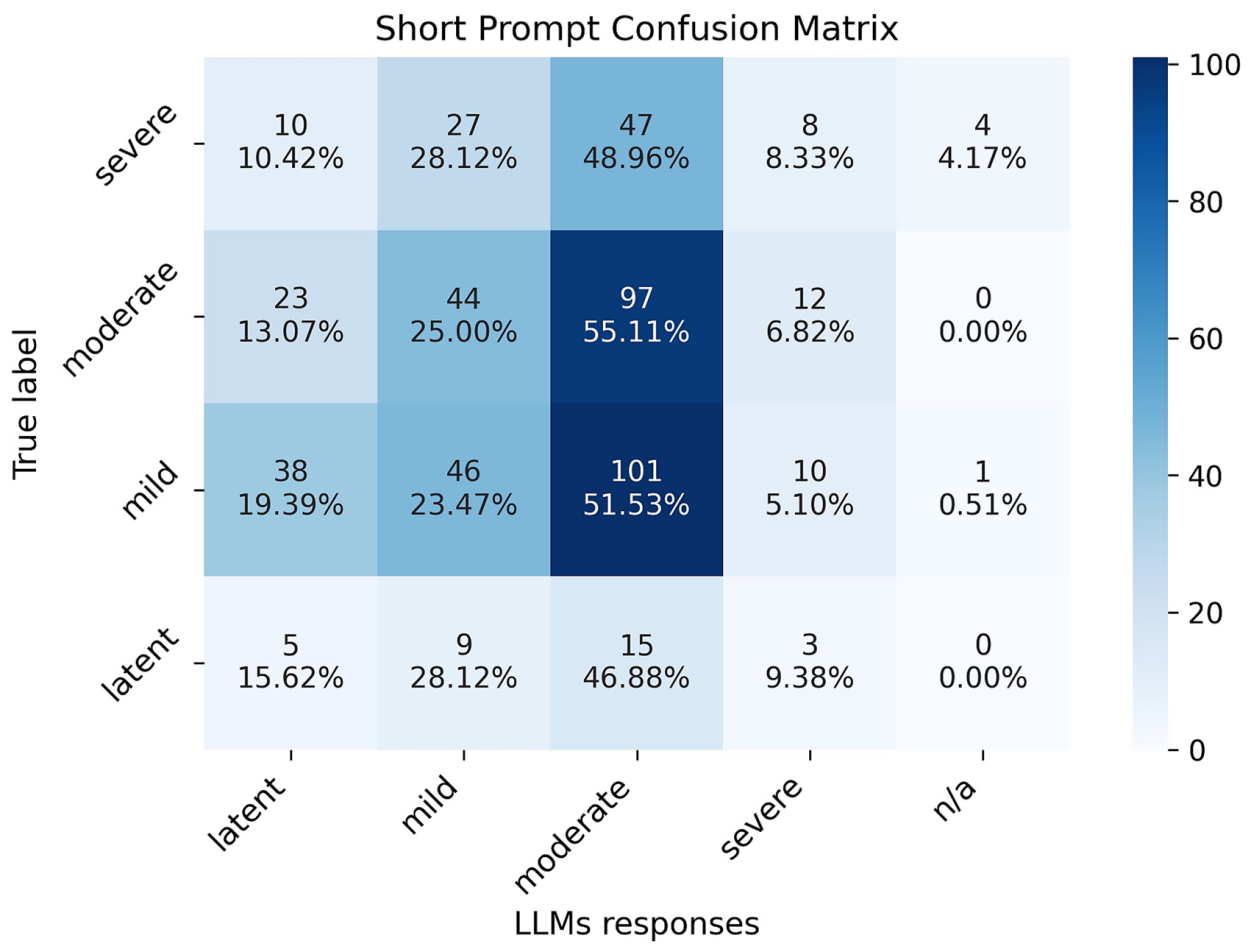
Confusion matrix for Llama-3.1-1B short prompt in detecting aphasia severity. N.A.: irrelevant LLM responses that do not fall into any of the four true labels.

In general, Study 3 results indicated that predicting severity via prompting (with various prompt ablation types) alone on a smaller parameter model is highly challenging, and the model defaults to a single class regardless of input. In particular, regarding short prompt (Figure 7), the model exhibited a strong bias toward the moderate class, predicting it 51.53% of the time for true mild cases and 55.11% for true moderate cases, effectively failing to distinguish between them.

**Figure 8:**
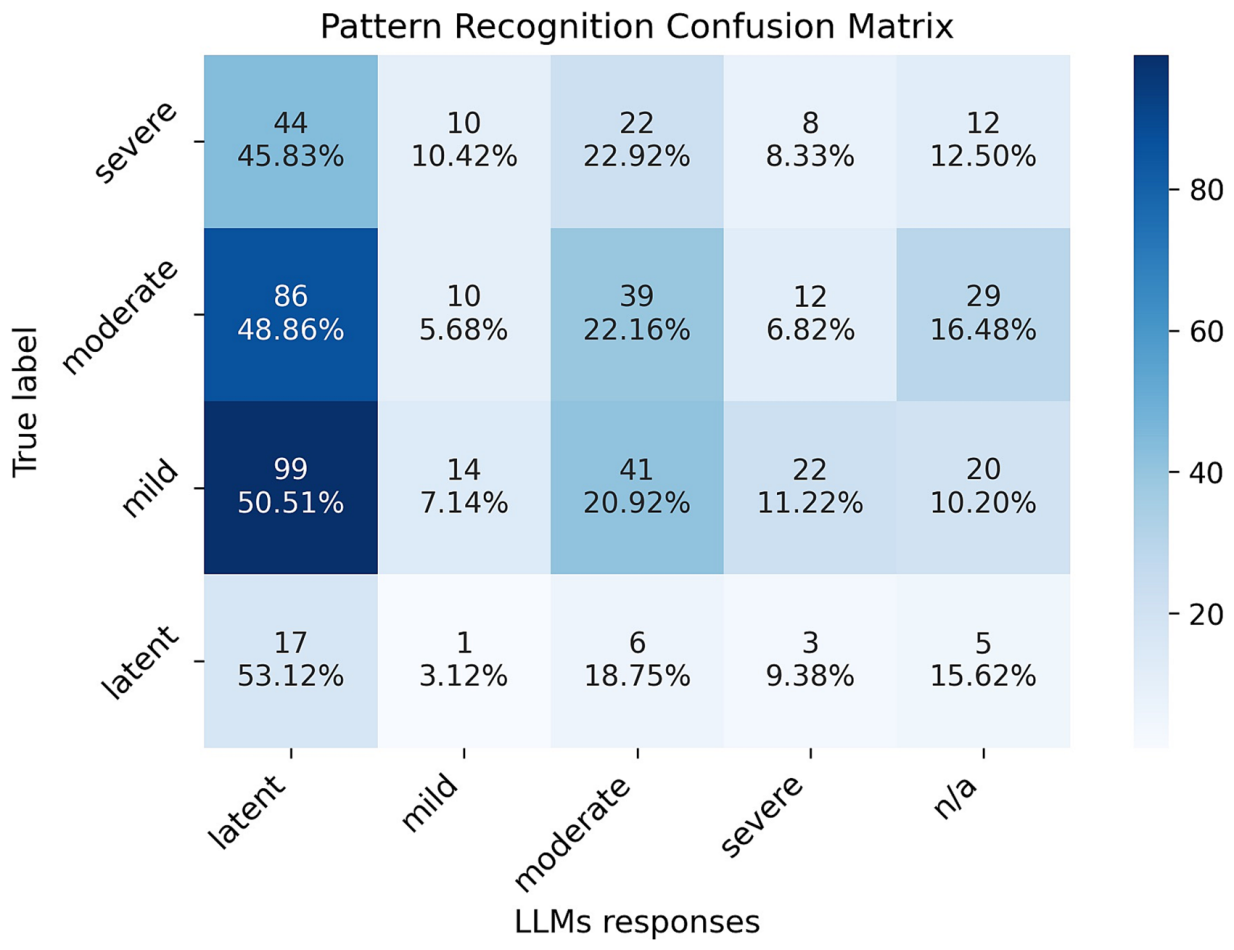
Confusion matrix for Llama-3.1-1B pattern recognition prompt in detecting aphasia severity. N.A.: irrelevant LLM responses that do not fall into any of the four true labels.

Regarding pattern recognition prompt (Figure 8), this strategy shifted the bias but did not improve accuracy, with the model over-predicting latent aphasia (53.12% of true latent cases identified) but failing to identify mild cases (only 7.14% correct).

**Figure 9:**
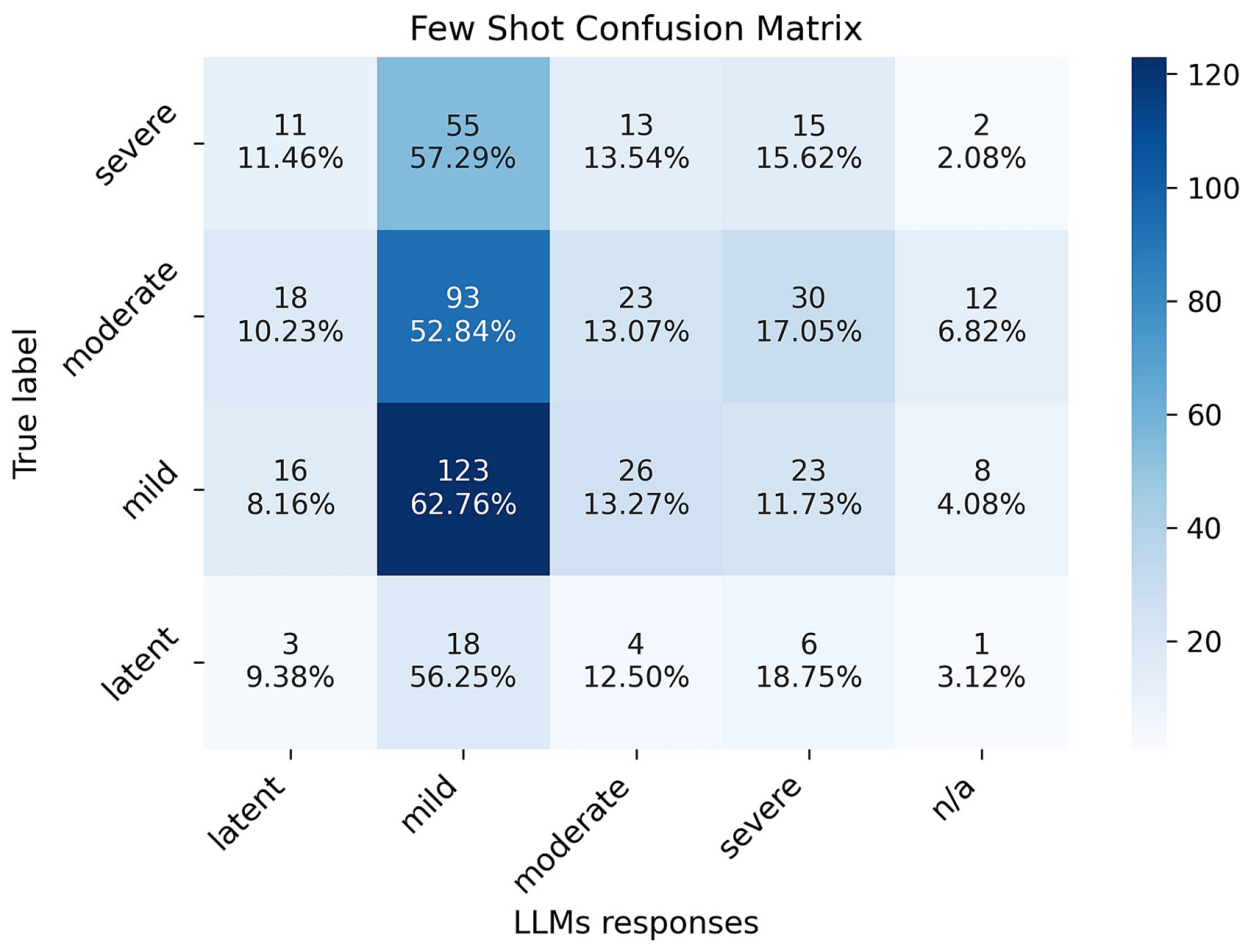
Confusion matrix for Llama-3.1-1B few-shot prompt in detecting aphasia severity. N.A.: irrelevant LLM responses that do not fall into any of the four true labels.

**Figure 10:**
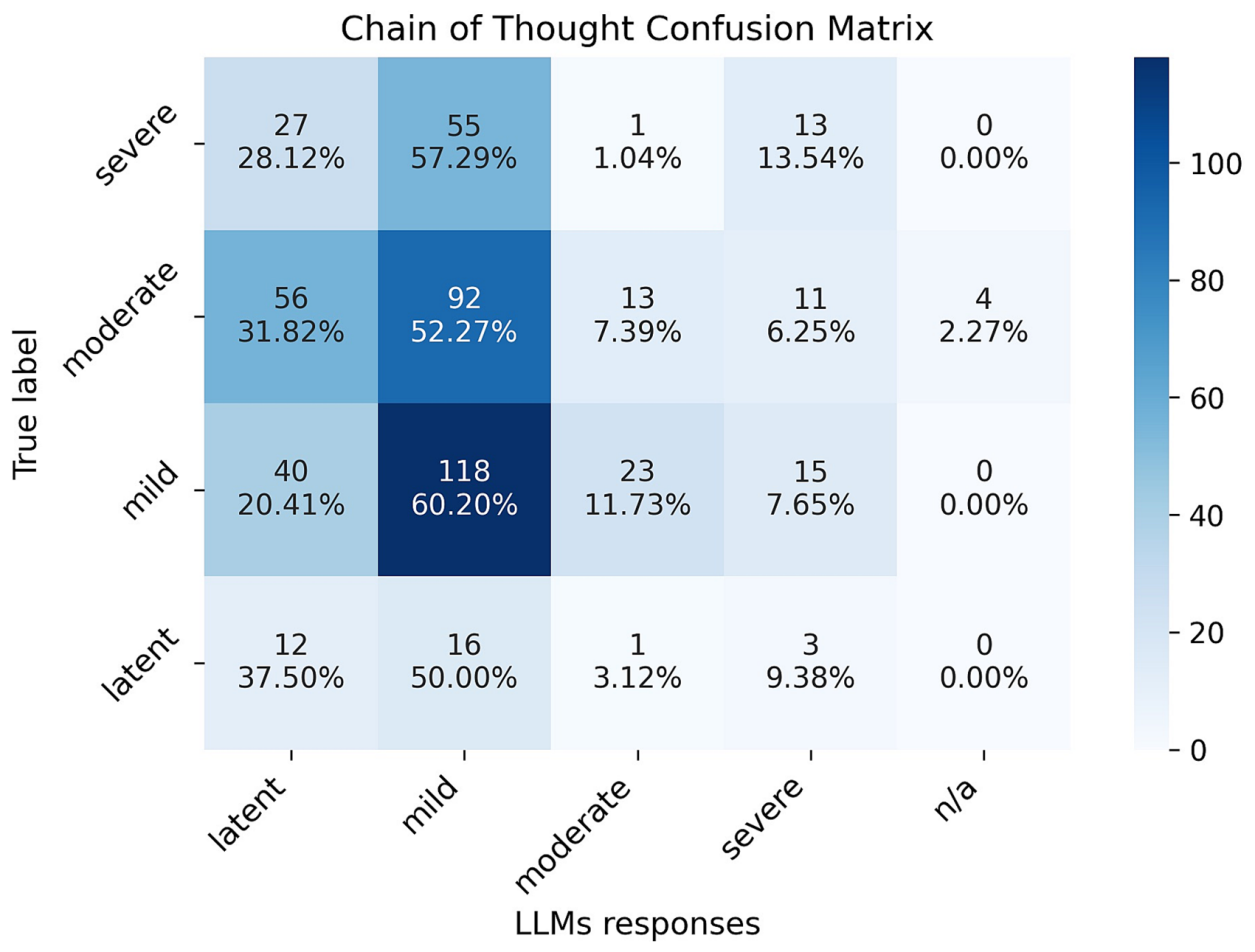
Confusion matrix for Llama-3.1-1B CoT prompt in detecting aphasia severity. N.A.: irrelevant LLM responses that do not fall into any of the four true labels.

Regarding few-shot and CoT (Figures 9, 10), introducing examples caused the model to over-generalize the mild label. In the few-shot condition (Figure 9), the model predicted mild for 62.76% of actual mild cases, but it incorrectly predicted mild for 56.70% of severe cases. Adding Chain-of-Thought reasoning did not mitigate this (Figure 10), resulting in 57.29% of severe cases still being misclassified as mild.

Overall, as shown in Table 6, the few-shot prompt performed the best (accuracy score = 0.328), having better accuracy in its predictions across all severity groups than the other prompts. Across prompting conditions, accuracy remained only slightly above chance (31–33%). All the prompts demonstrated tendencies to predicting primarily one severity, despite being provided conversations categorized across all severities of aphasia.

**Table 6:** Evaluation metrics for prompting LLM in severity classification.

Prompt ablation type	Accuracy	Precision	Recall	F1 score
Short	0.312	0.326	0.312	0.298
Pattern recognition	0.156	0.323	0.156	0.174
Few-shot	0.328	0.332	0.328	0.304
CoT	0.312	0.35	0.312	0.282

Qualitative error analysis revealed that while the model could identify some disfluencies and incoherent and irrelevant productions, it struggled to quantify them into discrete severity stages. For example, in successful cases, the model correctly identified repetition and unrelated speech as “Severe.” However, in unsuccessful cases, the model often hallucinated incoherence in mild patients or failed to detect the nuance of latent deficits. We further examined the direct output from the LLM, when its response is limited to a short paragraph. Below is the LLM response to a severe case (true label: severe):

*A conversation with a person with severe aphasia about floods:*

*Aphasia Severity: Severe.*

*The provided conversation is a dialogue between two people, Flood and Nan. In this conversation, Flood is repeating the same words and phrases and speaking about things not related to the topic of conversation. These characteristics are indicative of a severe aphasia.*

*The repetition of words and phrases, such as “oh” and “yeah,” suggests a significant loss of fluent speech production. The use of similar sounds or words to replace correct phrases, such as “one” and “nan,” further supports the diagnosis of aphasia.*

From the response, the LLM identified two patterns, repetition and off-topic speech (speech with content irrelevant to the topic). Although the LLM prediction in that conversation was successful, we can observe the LLM detecting irrelevant patterns in this incorrect prediction below:

*A conversation with a person with mild aphasia about a boy kicking a soccer ball:*

*Category: Aphasia (Severe).*

*Explanation: The conversation appears to be a simple exchange between two people, with the speaker using a repetitive and nonsensical phrase…The speaker uses similar sounds and words to replace correct phrases (“like” and “it” are replaced by “um”), and uses words that do not sound normal (“soccer” instead of “football”). The conversation is also characterized by a lack of coherence and a lack of context*…

This speech sample is labelled as mild aphasia (true label); however, the LLM misclassified it as severe aphasia. The LLM appears to misinterpret off-topic or atypical language, treating lexical variation (e.g., “soccer” vs. “football”) as a sign of impairment, when it may instead reflect differences in the speaker’s linguistic background (e.g., regional variation such as Europe vs. the U.S.) rather than aphasia. These findings suggest that while prompting strategies can elicit surface-level reasoning, they are insufficient for multi-class severity classification in smaller models. In contrast to off-the-shelf, pre-trained models, these results indicate that domain- and task-specific fine-tuning may be required to better capture the relationship between linguistic features and clinical severity scores. Our current work concerns *evaluating* prompting approaches, providing an initial step for future work toward *optimizing* model performance by finetuning on severity detection tasks. We discuss this in-depth in the Discussion section.

Results of the additional classification tasks predicting WAB-fluency categories were provided in the Supplementary material S6. Findings are suggestive of the use of WAB fluency as a potentially valid alternative to WAB-R-AQ. Future work could take this as a proof-of-concept for computational feasibility, systematically extending our approach to various WAB-R subdomain scores.

## 5 Discussion

We aimed to take an initial step toward developing a clinically predictive tool for language dysfunction assessment (such as in aphasia) by fine-tuning LLMs on real and synthetic aphasia data. With textual data extracted from the AphasiaBank, we focused on assessing sentence-level agrammatic features and an LLM-derived surprisal index of linguistic atypicality. Fine-tuning improved LLM performance on the surprisal-based classification task (Study 2, Task 2), but it did not yield consistent improvements over the logistic regression baseline for agrammatic utterance detection (Study 2, Task 1). We also highlighted methodological challenges in synthetic data generation and human evaluation of such data (Study 1). As a proof of concept, we conclude that while the fine-tuned models demonstrate promise in modeling aspects of language atypicality, these findings do not establish clinical validity, and further improvements in data quality and model design are needed before these approaches can be considered for clinical use. In what follows, we further discuss the implications of the results and future directions.

### 5.1 LLMs in psychological science

Our investigation contributes to ongoing efforts to evaluate the role of LLMs in psychological and language-related research by examining their capacity to simulate and classify patterns of language dysfunction. Specifically, we focused on how LLMs, when fine-tuned on both real and AI-generated synthetic data, perform in tasks related to detecting agrammatic utterances and broader aphasic-like language atypicalities, both of which are relevant to neuropsychological assessment and cognitive-linguistic profiling.

Results indicate that classic machine learning models, such logistic regression, remained strong for agrammatic utterance detection. In contrast, fine-tuned LLMs, particularly Llama, showed advantages on the surprisal-based classification task. Text-only synthetic augmentation appeared feasible and may support improved detection and generalization of aphasia-like speech when data are scarce. Our results also suggested that modern LLM architectures (dense transformer architecture as in Llama and MoE as in Gemini), along with pretraining data coverage, training regime, and other potential factors, may be related to LLMs’ ability to learn more complex linguistic patterns associated with language impairments. This finding was salient when data augmentation techniques are applied. Our current work does not definitively attribute such ability to any particular factor. Future studies could compare LLMs on the same axes, differing only in one factor (e.g., Llama-instruction-tuning versus Llama-base), to identify causal relationships of specific model properties and model performances.

Our results reveal both the promise and the limits of using LLMs. The ability of LLMs to outperform both baselines and pre-trained variants in a subset of our tasks suggests that there is meaningful information to be learned from fine-tuning. At the same time, inconsistencies in rater agreement and classification errors highlight the need for continued work in model interpretability, error analysis, and multimodal validation. This aligns with the broader goals of psychological science, to balance innovation in measurement with careful evaluation of validity and generalizability. While still exploratory, our study demonstrated a pathway for integrating LLMs into clinical language research, with potential implications for future assessment tools, data simulation pipelines, and models of language processing in broader clinical populations.

Our findings regarding the capabilities of LLMs must be contextualized within the growing debate on their cognitive validity, particularly the critical perspective that LLMs do not genuinely simulate human psychology (Schröder et al., 2025). While our results provide preliminary evidence that depending on tasks, models can generate and simulate surface-level linguistic features, this capacity should not be conflated with the replication of the underlying neurocognitive mechanisms driving aphasia. The mixed inter-rater reliability observed in our validation studies and the models’ difficulty in capturing subtle, context-dependent markers of dysfunction (i.e., agrammatic utterances) align with the view that LLMs likely operate as probabilistic mimics rather than psychological agents. Relatedly, the illustrative example wherein the model wrongly categorizes mild aphasia as severe due to its treatment of “soccer” as a lexically deviant form (presumably expecting “football”) serves as another demonstration for LLMs likely operating as probabilistic mimics. It shows that the model seems to be using superficial heuristics derived from its pre-training distribution rather than deep, real clinical reasoning. Overall, the LLMs we studied here do not seem to have the capacity of possessing true human psychology or multimodal grounding, and underscore that while LLMs are potentially clinically useful, they are not able to replace or supplant human domain experts at this time.

### 5.2 Synthetic data generation and validation

The scarcity of high-quality, individual-level clinical data raises a challenge in training and validating LLMs for aphasia assessment. With only approximately 300 participants in the archived AphasiaBank dataset, the alignment between LLM outputs and human expert assessments of aphasia remains limited by data availability. By approximating the linguistic manifestations of language dysfunction, LLMs offer a pragmatic solution to data scarcity, provided they are deployed as auxiliary instruments for data augmentation and hypothesis testing rather than as substitutes for human clinical reasoning.

Our synthetic data generation (Study 1) offers a potential solution by augmenting the training set and addressing sparsity, especially for rare or severe cases of aphasia that may not be well-represented in the original data. Comparing LLMs fine-tuned with and without AI-generated synthetic data highlights the potential utility of synthetic data. We summarize that synthetic data may introduce noise, but with careful validation, it may be able to improve model generalizability by amplifying linguistic patterns represented in the original dataset.

Our synthetic data validation (Study 1) yielded nuanced results, which highlight both the potential and the limitations of LLM-generated clinical data. In the web-based survey, raters identified the source of utterances at near-chance levels, resulting in a negative inter-rater agreement. While this could suggest a degree of surface-level fidelity, such results also likely reflect high rater uncertainty and task ambiguity inherent in judging isolated, text-only five-utterance snippets. Our current experiment and task design does not tease apart these possibilities, and this points to a core challenge. While LLMs can approximate certain surface-level agrammatic features, reproducing the subtle, context-dependent clinical markers of aphasia remains a complex task for current models, which perform these tasks as probabilistic mimics rather than true psychological agents. Our findings resonate with prior work (Salem et al., 2023), suggesting that synthetic data quality remains a challenging issue, although it can potentially provide significant utility for classification tasks when real data is scarce.

Validation remains critical to ensure that AI-generated synthetic data accurately reflects clinical phenomena and does not amplify biases or inaccuracies inherent in the base model or dataset. Concretely, validating synthetic data is crucial to ensure its fidelity, utility, and privacy (Azizi et al., 2021; Hu et al., 2023; Salem et al., 2023). Fidelity refers to how closely the synthetic data mirrors the original dataset’s statistical properties, while utility assesses its effectiveness for specific analytical tasks. Privacy ensures that the synthetic data does not reveal sensitive information from the original dataset. In this study, confusion matrices and inter-rater agreements in our primary web-based validation survey revealed that further research on fidelity, utility, and privacy is needed, before one can conclude that synthetic data accurately reflects the true characteristics of language dysfunction. Plus, further evaluations are necessary to assess utility and privacy comprehensively. Implementing a multi-faceted evaluation framework as shown in Hu et al. (2023), can provide a more thorough assessment by measuring these dimensions across various metrics. Additionally, adopting visual and statistical techniques can aid in assessing the fidelity of synthetic data. Interactive visualizations and statistical indicators can help determine whether the synthetic data retains the same statistical information, correlations, and properties as the original data. We leave these directions for future research.

### 5.3 Efficacy of fine-tuned LLMs

Different from the previous work where pre-trained LLMs were taken off the shelf and applied to clinical tasks, here we tailored LLMs through fine-tuning on aphasia datasets. Our findings imply that fine-tuned LLMs represent a promising step toward automating aspects of clinical aphasia assessment, but their clinical efficacy depends on their ability to mimic human-like diagnostic reasoning. Unlike clinicians, who assess discourse holistically across extended sessions, current LLMs in our study processed text line-by-line, which may limit their capacity for nuanced analysis of narrative coherence or agrammatic tendencies. While the strategy of using a five-utterance context window can alleviate this concern, it might not completely address it: limitations in input size (e.g., 5–10 utterances or one short narrative) might constrain the models’ ability to reflect real-world diagnostic contexts, which are inherently multimodal, whereas our current pipeline does not consider non-textual cues such as prosody, facial expressions, and gestures. Our supplementary, additional experiment with a three-utterance context window provided preliminary evidence that small input size affects models’ performance. Also, the lack of access to multimodal data likely explains the SLPs’ and trained RAs’ rating behaviors in the web-based online survey. Consequently, while these models provide insights, their use in clinical workflows requires significant advancements for these models to approximate the holistic and multimodal reasoning of human clinicians.

A striking finding of Study 2 is that the logistic regression baseline outperformed both fine-tuned Llama and Gemini models in the detection of agrammatic utterances (Task 1). One possible explanation is that the linguistic signal for agrammatic utterances in our dataset seems largely surface-transparent, in that it can be identified from explicit surface-level features without requiring broader contextual interpretation. Classic feature engineering is particularly well-suited for such patterns. The TF-IDF mechanism effectively reveals the rare, broken constructions and missing words typical of agrammatic speech, allowing a linear model to achieve high discriminative power without the need for the massive parameter space of a transformer LLM. In these instances, the added complexity of LLMs might not necessarily bring in benefits, as the local *n*-gram features capture the primary diagnostic markers of the condition more efficiently. Overall, classical models might remain a go-to choice for well-defined linguistic markers. It depends on task design whether or not (fine-tuned) LLMs can offer a complementary, high-resolution tool for capturing complex atypical language production. Our Study 2 showed proof-of-concept that LLM may outperform linear models on the surprisal-based classification task, suggesting improved sensitivity to distributed patterns of linguistic atypicality. Future studies could implement this pipeline on more systematic tasks, broader clinical populations, and larger datasets.

It is also worth highlighting that, in Study 2, although fine-tuned models might seem promising as a final product, and fine-tuning is significantly lighter than the original pre-training phase, fine-tuning is still computationally expensive, relative to other LLM-related tasks such as inferencing (as in Study 3), where users can deploy a pre-trained LLM and apply it directly to aphasia assessment tasks, without parameter tuning and additional training (Tunstall et al., 2022; Jurafsky and Martin, 2025). This might pose a challenge to aphasia researchers and clinicians with limited computational resources. Here we provided a showcase of LLMs fine-tuning on augmented datasets, a step toward our ultimate goal of providing a fine-tuned LLM for the aphasia research community, such that the end users can utilize a tailored aphasia-specific LLM in daily routine.

### 5.4 Labels: machine processing versus human expert assessment

In the present study, we used archived data provided by the AphasiaBank corpus, a valuable resource for prototyping and exploring computational approaches such as fine-tuning LLMs on real and augmented aphasia data. AphasiaBank is particularly useful due to its computer-friendly and relatively consistent annotations for certain linguistic features, which provide meaningful labels for classification tasks in our machine-related investigation.

For example, the label about agrammatic speech [+ gram] was used in tuning and teaching LLMs, helping the models learn to differentiate grammatical and ungrammatical utterances. However, agrammatic utterance is not a single, uniform condition but rather a constellation of symptoms that can vary in their presentation depending on the severity of the impairment and the specific language functions affected. Therefore, clinical testing often aims to determine the extent to which grammatical elements are missing or incorrectly produced, which is a complex, multi-faceted process that operates more effectively on a gradient, rather than being reduced to a binary categorical label, like we used in this study. While we incorporated multiple agrammatic features into our fine-tuning pipeline, these machine-friendly features might be an oversimplification compared to human experts. It seems an obvious oversimplification to directly connect labels such as [+ gram] to the complex condition of agrammatic utterances in aphasia. We maintain that such a label provides a starting point for machine processing, particularly for exploring the use of computational methods in understanding, diagnosing, and simulating speech impairments. The ability to categorize utterances as [+ gram] or [− gram] based on AphasiaBank data offers a simplified, yet useful, framework for testing hypotheses and refining machine learning and LLMs techniques in aphasia assessment, such as agrammatic speech.

Besides the agrammatic label, the LLM-surprisal based index label is worth in-depth discussion. It is derived by computing GPT-2 surprisal scores over utterances extracted from AphasiaBank. This label is not a clinical construct but rather a computational quantity, specifically a binarized summary statistic obtained from a different language model (GPT-2). When evaluating Llama and Gemini on their ability to predict this label, they are essentially being asked to approximate GPT-2’s surprisal function rather than directly detecting aphasia in the traditional sense. Thus, we emphasize that Task 2 serves as a computational demonstration aimed at comparing the performance of LLMs and linear baselines (logistic regression) in capturing the intricate, non-linear linguistic patterns that contribute to high surprisal in disordered speech. In other words, we highlight that this computational demonstration does not provide a definitive clinical validation; instead, one likely interpretation of the ability of one transformer to predict another’s surprisal index concerns their shared architectural priors. While we maintain that LLM-surprisal serves as a valid proxy for atypical language production, supported by its correlation with clinical markers in previous research (Rezaii et al., 2023; Cong et al., 2024a, 2024b), we recognize that these findings constitute a proof-of-concept for probabilistic, data-driven modeling of clinical signals rather than a substitute for clinical diagnosis, providing pipelines and methodological aspects for evaluating how models learn to identify broader profiles of speech atypicalities.

Looking toward future work, we aim to incorporate laboratory-based structured data, wherein certified clinicians and aphasia researchers systematically provide more precise and accurate labels of different agrammatic features. This would allow us to improve the training and generalization capabilities of machine learning algorithms, ultimately leading to more accurate and clinically useful tools for diagnosing and treating language disorders. By combining clinical expertise with computational analysis, we hope to enhance both the precision of classification tasks and the development of more effective interventions.

### 5.5 Prompting approaches reveal clinical blind spots of LLMs

Our exploratory analysis in Study 3 demonstrated that end-to-end aphasia severity classification via prompting alone is highly challenging for a smaller-parameter LLM. This exploratory analysis reveals limitations of prompt-based approaches and highlights the importance of task-specific fine-tuning and richer clinical linguistic representations. Across prompt ablation conditions, Llama-3.1-1B showed strong class biases and limited discriminative ability, frequently defaulting to a single severity label regardless of input. Short prompts biased predictions toward the moderate class, pattern-recognition prompts shifted the bias toward latent aphasia without improving accuracy, and few-shot (+CoT) prompting induced over-generalization of the mild label, leading to substantial misclassification of severe cases. Although few-shot prompting yielded the highest overall accuracy, performance remained low across all conditions. Qualitative error analysis indicated that while the model could detect surface-level disfluencies (e.g., repetitions, off-topic productions), it struggled to map these features onto clinically meaningful severity distinctions and occasionally hallucinated impairments or misinterpreted lexical choices.

From a theoretical perspective, Study 3 findings are not entirely unexpected. Notably, applying the session-level WAB-R AQ score to individual utterances serves as a categorical approximation. This approach might introduce noise to model, as a patient labeled “mild” at the aggregate level may produce specific utterances that span the severity spectrum during a single conversation. These labels should therefore be cautiously interpreted as indicators of the speaker’s broader clinical profile rather than absolute utterance-level ground truth. Also, future studies could adopt other clinical metrics and more fine-grained WAB-R subdomain scores such as WAB-R fluency ratings. Although the analyses incorporated multiple linguistic indicators, aphasia severity, as operationalized by WAB-R scores, emerges from the interaction of diverse communicative dimensions, including lexical, syntactic, semantic, and functional abilities. Capturing this multidimensional construct through prompting alone places substantial demands on smaller LLMs, particularly given the heterogeneity of aphasia presentations and the subtle changes between severity levels. Beyond computational constraints and sample distribution factors, the limited predictability likely reflects the inherent difficulty of mapping complex clinical constructs from discourse-level language data. More broadly, these results highlight a key limitation of general-purpose AI models in clinical inference: surface-level pattern recognition does not necessarily translate directly into reliable severity staging.

Lastly, Study 3 included an exploratory examination of subclinical populations in aphasia, for example, latent aphasia. This allowed us to reveal *some* of LLM’s efficacy in assisting the severity detection for this subclinical population. Latent aphasia, a subtle and often underdiagnosed condition, requires improved diagnostic tools and refined metrics due to its nuanced presentation, such as increased pause durations and lexical retrieval difficulties in discourse (DeDe and Salis, 2020; Fromm et al., 2017; Silkes et al., 2021). Currently, the lack of sufficient data for latent aphasia limits model development and fine-tuning. Expanding the dataset with high-quality AI-generated synthetic data and refining LLMs to better predict language features, such as word-finding behaviors and discourse production patterns, will be crucial to further understanding this subclinical population. We maintain that future directions for fine-tuning LLMs and synthetic data generation in aphasia research could implement our pipeline and approach, focusing on addressing the challenges posed by the small latent aphasia dataset.

### 5.6 Ethical considerations

This work raises important ethical considerations regarding the use of AI-generated synthetic data in clinical research. Synthetic data generation necessarily reflects patterns present in the underlying training data and may therefore reproduce or amplify existing demographic and linguistic biases (Bedrick et al., 2025). Moreover, the discussion of representation bias in synthetic data extends beyond demographic representation, including dialectal and sociolectal variation. Synthetic data in our study may not necessarily be immediately generalizable to other varieties of English or to speakers whose baseline language production already deviates from the assumed “norm.” In addition, while automated language models may support research workflows, they should not be interpreted as clinical tools or deployed in assessment contexts without extensive validation, as errors could have meaningful consequences for patient care. Finally, although synthetic data may mitigate some privacy concerns, the human validation procedures in this study relied on real patient utterances drawn from datasets collected with appropriate informed consent and IRB approval. These considerations highlight the need for cautious interpretation, transparent reporting, and responsible use of LLM-based methods in clinical research.

## 6 Limitations

There are several limitations of our current work. First, our work does not address multimodality data. The reliance on text-only data excludes critical multimodal elements, such as audio or visual cues, which are central to clinical aphasia diagnosis. However, the lack of open-source, multimodal generative models hinders the use of such multimodal data, ultimately limiting the broader adoption of LLMs to clinical contexts. Moreover, there are significantly fewer reliable multimodal corpora for aphasia speech, which involve well-annotated video, image, sound, and text (e.g., AphasiaBank). Current generative LLMs also struggle to handle sparse or overly cleaned and pre-processed datasets, which may lack the variability necessary to train robust models. We focused on simulating agrammatic utterances in this study; however, future research could expand to simulate a broader profile of aphasia. We acknowledge that incorporating longer contexts or leveraging multimodal LLMs may help simulate broader profiles of aphasia. In particular, multimodal LLMs could play a critical role in generating more ecologically valid profiles of aphasia. Aphasia is not only expressed through linguistic content (e.g., grammar and word retrieval) but also through prosody, articulation, and gesture. By integrating text, speech, and visual modalities, multimodal models could simulate how individuals with aphasia combine or struggle to combine these communicative channels in naturalistic contexts. For example, such models could produce utterances that capture both linguistic deficits (e.g., agrammatic utterances, paraphasias) and accompanying nonverbal behaviors (e.g., pausing, gestures). This would enable a richer representation of how aphasia manifests in real-world communication, beyond isolated utterances.

Moreover, multimodal simulation could support research into clinician and caregiver perception: raters could be presented not only with text or audio but also with audiovisual scenarios that more closely mirror clinical interactions. This may improve ecological validity and allow more reliable evaluation of whether AI-generated data faithfully reflects the complexity of aphasia. Because aphasia is a multifaceted clinical condition, it remains inherently difficult to simulate and judge at the utterance level, particularly in the absence of contextual or diagnostic cues. Future research should therefore explore multimodal methods that provide richer linguistic, prosodic, and contextual signals, alongside diagnostic frameworks, to better capture and assess the complexity of aphasia.

Second, the human validation study is of limited scale. We acknowledge that the SLP rating experiment had a small sample size, and we included both certified SLPs and trained student RAs. We leave larger-scale, fully controlled certified SLP validation experiments for future research. We maintain that our pipeline should generalize. We argue that the current sample size is appropriate for the study’s scope and aligns with recent methodologies. Our validation survey design was inspired by Salem et al. (2023), which established a pipeline for initial validation in aphasia research. Also, we implemented computational analyses, reported 95% Confidence Intervals (CIs) for our performance metrics, and conducted significance testing (e.g., McNemar’s test) to ensure our findings are statistically robust without requiring additional human subject recruitment. Future research could explore larger scales, with more certified SLP participants and more items, so that systematic investigation of individual participant-level or item-level analysis could be conducted. This study focuses on evaluating the computational utility of LLMs for data augmentation and simulation. As noted, this study is a proof-of-concept to demonstrate that synthetic data can approximate surface-level agrammatic features. Conducting a larger-scale human validation study with over 50 certified SLP raters, with similar clinical experience and training (addressing the potential confound of the heterogeneity of rater expertise), on over 1,000 items would shift the focus to clinical instrument validation, which is outside the scope of this computational investigation. Future work should include full validation with certified SLPs, ideally conducted within clinical and aphasia community settings to ensure ecological validity and clinical relevance.

The inclusion of both SLPs and trained student RAs may have introduced variance in the ratings. Although inferential statistics showed that identification accuracy did not differ significantly between clinical experts and students, future studies could invite more participants, with pre-registered clinical expertise documentation. As this study is a proof-of-concept focused on the computational utility of synthetic data for model training rather than the validation of a clinical diagnostic tool, we maintain that this initial heterogenous sample provides a useful, albeit preliminary, baseline for data quality. Notably, raters who are informed that the survey includes both agrammatic and non-agrammatic items may adopt response strategies that either inflate or deflate the marginal response rates. A larger pool of raters could potentially mitigate this factor. We acknowledge the cautious interpretation of validation findings throughout the manuscript. Overall, future research should utilize larger, stratified samples of certified clinicians to isolate the impact of clinical experience (among other rater-related factors) on the detection of synthetic aphasic speech.

Third, our work has methodological limitations. Our study utilized a fixed window size of five utterances, with an additional, supplementary analysis on a window size of three utterances. While this size was chosen for its alignment with narrative units in the Cinderella and picture description tasks, we acknowledge that window size is a hyperparameter that may influence classification accuracy. Future research should conduct systematic ablation studies to determine how varying context lengths (e.g., 3 vs. 7 vs. 10 vs. 20 vs. 35 utterances) affect a model’s ability to capture long-range dependencies versus local grammatical errors in aphasic speech.

We also emphasize that studies involving prompt engineering require larger sample sizes and more systematic manipulation of prompts to ensure reproducibility. For example, future research could conduct controlled experiments to examine how different prompt variants using different AI models statistically influence the quality of synthetic data. In our current study on synthetic data generation using GPT-4o-mini, the selection of effective prompts relied on manual expert judgment together with the AphasiaBank protocols and examples guidance. Without an automated or quantitative metric, our decision process was essentially subjective and non-reproducible. This approach served as a necessary preliminary sanity check. As automated discourse analysis methods continue to develop, systematically integrating and reproducing these elements will be essential for creating clinically meaningful and generalizable tools for aphasia assessment.

Moreover, we acknowledge that although fine-tuning improved classification F1 scores, the small test set for Task 1 (37 snippets per class) means these specific findings should be viewed as preliminary. In contrast, our Task 2 analysis (LLM-surprisal indexed utterance detection) utilized a larger dataset (600 snippets), allowing for more robust statistical conclusions via McNemar’s test.

Lastly, a limitation of our cross-model comparisons in Task 2 concerns the divergent fine-tuning regimes applied to Llama and Gemini. Due to API and accessibility constraints, the open-source LLM Llama was fine-tuned using Parameter-Efficient Fine-Tuning (PEFT) with LoRA. In contrast, the commercial, close-source LLM Gemini was subject to a standard Supervised Fine-Tuning protocol via the provider’s API. These two mechanisms differ fundamentally in their regularization profiles, the proportion of model parameters modified, and their resulting adaptation to domain-specific data. Consequently, the performance gap observed between the two models, where Llama achieved higher F1 scores than Gemini in LLM-surprisal index utterance detection, cannot be definitively attributed to one factor (e.g., dense transformer vs. Mixture-of-Experts) alone. It is possible that the different training conditions themselves influenced the models’ ability to capture the complex linguistic markers of aphasia. Future research on unified fine-tuning environments on open-source weights could isolate factors such as architectural efficacy and training methodology.

## 7 Conclusion

Our work explored how LLMs can be leveraged to both generate and classify disordered language, with a focus on aphasia. Our findings provide proof-of-concept that synthetic utterances produced by LLMs can approximate surface-level features of agrammatic utterances, and they have the potential to support downstream tasks. Moreover, transformer-based models, particularly when fine-tuned with curated synthetic data, demonstrated the ability to detect meaningful patterns of language dysfunction in a subset of our tasks. Specifically, while models excelled at learning the statistical atypicality indexed by LLM-surprisal, this likely reflects a capacity for (inter-LLM) pattern approximation; more systematic research is needed to provide solid evidence that the identification of clinical constructs like agrammatism can be more effectively handled by LLMs than by simpler linear baselines. These preliminary and suggestive results highlight promising avenues for augmenting limited clinical datasets and for probing the linguistic structure of disordered speech; crucially, they also emphasize the need for caution. The apparent validity of synthetic data does not guarantee fidelity to the full complexity of aphasic language, and further work is needed to evaluate validity, generalizability and clinical relevance. Overall, our study suggests that LLMs may serve as valuable tools for both data augmentation and linguistic analysis in the study of language dysfunction, provided datasets are of sufficient quality and quantity, experiments are controlled, and limitations are acknowledged.

## Data Availability

The original contributions presented in the study are included in the article/Supplementary material, further inquiries can be directed to the corresponding author.

## Footnotes

1
https://huggingface.co/models

2
https://ai.google.dev/api

3
https://talkbank.org/aphasia/examples/index.html
