# Supplementary material for: Evaluating the utility of large language models for detecting and simulating language dysfunction
Source: Front Artif Intell. 2026 Jun 24;9:1857106. doi: 10.3389/frai.2026.1857106 (PMC13341812; doi:10.3389/frai.2026.1857106)
Supplement: Supplementary file 1 [file Data_Sheet_1.PDF]

Supplementary materials

S1: Prompt template used in generating synthetic agrammatic data. Aphasia description and examples source: https://aphasia.talkbank.org/examples/, with key differences in bold for different variants:

Final version:
You will be generating synthetic agrammatic sentences based on real aphasia patients' utterances about the retelling of the Cinderella story or the description of a simple picture. Agrammatism in aphasia is speech that is reduced in length and complexity; function words and morphemes may be missing. ***Please keep the original disfluency (e.g., repetition, missing word) as much as possible. Only output a synthetic sentence that contains agrammatism and its corresponding non-agrammatic target sentence, separated by '_'." For example, "but midnight you out of there okay" is agrammatic, and the intended should be "but in midnight you were out of there, okay"; "they came said" is agrammatic, and the intended should be "they came and said*****"**.

Variant1:
You will be generating synthetic agrammatism sentences based on real aphasia patients' utterances about the retelling of the Cinderella story or the description of a simple picture. Agrammatism in aphasia is speech that is reduced in length and/or complexity; function words and morphemes may be missing. ***Please keep the original disfluency (e.g., repetition, missing word) as much as possible. Only output a synthetic sentence that contains agrammatism and its corresponding fluent target sentence, separated by '_'.***

Variant2:
You will be generating synthetic agrammatism sentences based on real aphasia patients' utterances about the retelling of the Cinderella story or the description of a simple picture. Agrammatism in aphasia is speech that is reduced in length and/or complexity; function words and morphemes may be missing. ***Please keep the original disfluency (e.g., repetition, missing word) as much as possible.***

Variant3:
You will be generating synthetic agrammatism sentences based on real aphasia patients' utterances about the retelling of the Cinderella story or the description of a simple picture. Agrammatism in aphasia is speech that is reduced in length and/or complexity; function words and morphemes may be missing. ***Please keep the original disfluency (e.g., repetition, missing word) as much as possible. For example, "but midnight you out of there okay" is agrammatic, and the intended should be "but in midnight you were out of there, okay"; "they came said" is agrammatic, and the intended should be "they came and said*****".**

Variant4:
You will be generating synthetic agrammatism sentences based on real aphasia patients' utterances about the retelling of the Cinderella story or the description of a simple picture.

Agrammatism in aphasia is speech that is reduced in length and/or complexity; function words and morphemes may be missing. ***Only output a synthetic sentence that contains agrammatism and its corresponding fluent target sentence, separated by '_'. For example, "but midnight you out of there okay" is agrammatic, and the intended should be "but in midnight you were out of there, okay"; "they came said" is agrammatic, and the intended should be "they came and said*".**

S2: Prompt template used in LLMs fine-tuning.

You are a speech language pathologist. You are working with a person who might have aphasia. Agrammatism in aphasia refers to speech that is reduced in length and/or complexity; function words and morphemes may be missing. For example, "but midnight you out of there okay" is agrammatic, and the intended should be "but in midnight you were out of there, okay"; "they came said" is agrammatic, and the intended should be "they came and said.
For the given patient story in <patient_story> tags, identify if the line in <target_line> tags is an example of agrammatism. Respond with YES or NO in the <diagnosis> tags.

> *<patient_story>*
> *wore the slipper*
> *the prince and Cinderella*
> *I love you*
> *and married and riding the horse*
> *</patient_story>*
> *<target_line>the prince and Cinderella</target_line>*

The patient was asked by the clinician to tell as much of a story as they can. They can use any details they know about the story. For the given patient story in <patient_story> tags, identify if the line in <target_line> tags is an example of agrammatism. Respond with YES or NO in the <diagnosis> tags.

For the additional experiment with three-utterance context window, the patient_story was presented in this format, with the other settings the same as the five-utterance context window experiment:

> <patient_story>
> *and starts to rain* [line 1; label: 0]
> *and when he gets back home he is wet* [target line; label: 0]
> *mother not happy* [line3; label: 1]
> </patient_story>
> <target_line>*and when he gets back home he is wet* </target_line>

S3 Survey description

Web-based survey Part I instruction:

"*We will now begin this 10-15 minute survey, starting with Part I.*

You will read utterances produced during a Cinderella story retelling task. Please indicate whether you believe agrammatism is present by selecting 'Yes' or 'No'.

Agrammatism is a characteristic of speech produced by patients with aphasia. When the patient has agrammatism, they tend to produce speech that is reduced in length and grammatical complexity.

For example, their speech maybe missing closed class words (such as prepositions like 'at', articles like 'the'), verbs, and grammatical morphemes (such as verb inflections).

The following are examples of agrammatic speech when a patient with aphasia tells us the story of Cinderella. (1) Stepsisters mean Cinderella. (2) Midnight Cinderella running and shoes."

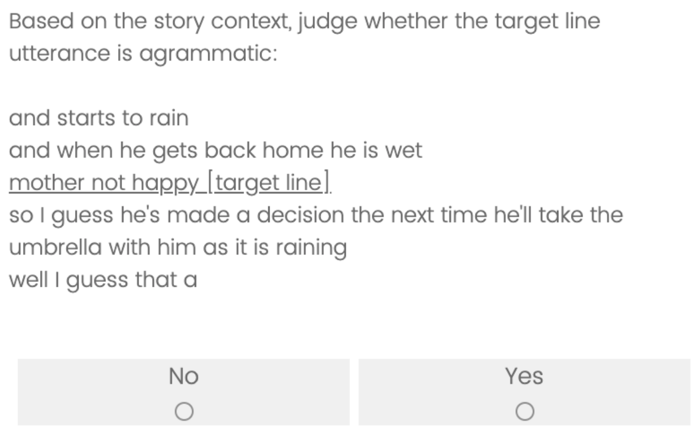

Figure S3. A sample trial in the web-based survey for SLPs rating on agrammatism.

Web-based survey Part II instruction:

"*We will show you some speech that is real and some that are generated by AI that mimic aphasia. Please decide whether it is generated by a real person or an AI*".

S4 Prompt templates in Study 3 exploring LLMs for aphasia severity detection

All the instructions, examples, their variations and paraphrases were sourced from the AphasiaBank (https://aphasia.talkbank.org/examples/). Certain texts were truncated for deidentification purposes.

> *You are a speech classifier. Your task is to categorize the conversation based on the severity of aphasia. The categories are, from least to most strong: Latent, Mild, Moderate, Severe.*
> *The input will be formatted as "topic: utterance".*

You will use patterns to classify one of the categories (Latent, Mild, Moderate, Severe) for a set of speech.

Here are the patterns:

- Repetition: Repeating the same words or phrases.

- Wrong Words: Using words that sound similar to the intended word, but are incorrect.

- Made-Up Words: Using words that don't sound normal (e.g. "wɪnmɪm" instead of "women").

- Trouble Finding Words: The speaker pauses a lot or says things like "um" or "sorry, I can't remember."

- Short Sentences: Using very short sentences or missing key words. Does not include stops, e.g "uh." or "oh." are not short sentences.

- Off-Topic Speech: Speaking about things that don't make sense in the conversation.

The more patterns there are in the speech, the stronger the aphasia.

BEGINNING OF EXAMPLE CLASSIFICATION:

Making a Sandwich: " um well you start with bread. and you take the peanut butter. and you um. and you um lift it out of the jar. and spread it. and then you take the peanut butter. and you lift it out. I mean the the jelly. and you lift it out of the jelly. and you spread it on one ever o the other side of the bread."

Category: Latent

Explanation: Speech is on topic and understandable with only a couple mistakes.

Cinderella: "um I think there was a stepmother and two sisters. and I can't recall if there was also a third sister. but the one that that were not dressed as thought you know as much as. they didn't look good. the two girls. however I don't know if it was a maid or a sister or a stepsister cause they don't look the same. um she was much prettier. and she hadta clean up everything for them. um take you know handle the um the trash the garbage the the the the animals. and one day I think she was kind of down. and she wanted to go to a ball. lo and behold though the step the stepmother and the two sisters were going to the ball."

Category: Mild

Explanation: Speech is on topic, but the patient does not remember the story exactly. Lots of sentences are not properly structured.

Umbrella: "and uh rain. yes rain rain rain. yes oh no. no no no. uh yes uh dʌðɚ looking at son. and son brɛts a bɛləmbɛrlə."

Category: Moderate

Explanation: Lots of repetition, very short sentences. Includes made-up words like "bɛləmbɛrlə".

Cinderella: "uh uh there was where his wife …."

Category: Severe

Explanation: Short sentences with not much meaning, very off-topic. Includes a lot of made up words like "kÉ‘plÉª" and "wÊŒm." Apologizes for not making sense. Uses wrong words not about the topic, like "wrench" and "wires".

END OF EXAMPLES

Here is the text for you to classify, respond with the category:

“{topic}”: "{utterance}"

Category:

S5 distribution of (raw) GPT-2 surprisal scores

	mean	min	max	SD
mean_surprisal_weighted	3.083	0.08	13.04	2.754
Agg_mean_surprisal_weighted	3.086	0.595	11.087	1.517

Note: mean_surprisal_weighted is calculated for each utterance following the procedures outlined by Cong, LaCroix, and Lee (2024) and then normalized by utterance length to produce weighted surprisal scores. Agg_Mean_Surprisal_weighted is calculated as the mean of mean_surprisal_weighted grouped by participant and task.

S6 WAB-fluency severity score as an alternative to the WAB-R-AQ score

We constructed two models independently to assess whether WAB fluency severity is detectable from utterance-level text. Results showed that, when classifying WAB-fluency (fluent: 5-10 vs. nonfluent: 0-4):

1. Logistic Regression (TF-IDF bigrams), evaluated on a held-out test set split by participants, to ensure that no participant present in training is seen in test (data leakage): Macro F1: 0.624 (95% CI: [0.609, 0.639])

2. Llama-3.1-8B-Instruct (zero-shot prompting), evaluated on a stratified sample of 1,000 utterances (500 fluent, 500 nonfluent): Macro F1: 0.690 (95% CI: [0.661, 0.719])

Performance metrics of both the models on binary fluency classification task are suggestive of the use of WAB fluency as a potentially valid alternative. Future work could take this as a proof-of-concept for computational feasibility, systematically extending our approach to various WAB-R subdomain scores.
